# How attributions of coproduction motives shape customer relationships over time

**DOI:** 10.1007/s11747-022-00910-6

**Published:** 2023-01-14

**Authors:** Pascal Güntürkün, Till Haumann, Laura Marie Edinger-Schons, Jan Wieseke

**Affiliations:** 1 Department of Marketing, Vienna University of Economics and Business, Welthandelsplatz 1, 1020 Vienna, Austria; 2 South Westphalia University of Applied Sciences, Lübecker Ring 2, 59494 Soest, Germany; 3 University of Hamburg, Rentzelstraße 7, 20146 Hamburg, Germany; 4 Ruhr-University of Bochum, Universitaetsstrasse 150, 44801 Bochum, Germany

**Keywords:** Customer participation, Coproduction, Inferred firm motives, Longitudinal design, Latent growth modeling

## Abstract

Despite the proliferation of coproduction concepts in various B2C contexts, knowledge on how coproduction shapes customer relationships is still surprisingly limited, as prior studies find mixed results and are bound to a short-term perspective. The present study addresses these limitations by providing first insights into the underlying psychological processes that explain differences in the short- and long-term relationship consequences of positive and negative coproduction perceptions. Drawing from the multiple inference model, this research shows how customers’ ambivalent attributions of a firm’s coproduction motives (i.e., firm-serving and customer-serving) affect customer satisfaction, willingness to pay, and spending behavior over time. The results of a latent growth analysis based on a longitudinal field study (n_1_ = 12,662; six waves) show that coproduction can harm customer relationships in the long-run, as the detrimental effects of firm-serving motive attributions are temporally more persistent than the favorable but ephemeral effects of customer-serving motive attributions. An additional experiment (n_2_ = 931) and field study (n_3_ = 360) confirm the generalizability of the key findings and provide new managerial insights into how firm-specific characteristics of a coproduction concept (i.e., coproduction intensity, design freedom, monetary savings) influence customer attributions different coproduction motives and thereby shape customer relationships over time.

**Supplementary Information:**

The online version contains supplementary material available at 10.1007/s11747-022-00910-6.

Whether in banking, retailing, traveling or e-commerce, B2C firms are increasingly relying on coproduction concepts in which consumers actively participate in the production of goods and services without direct assistance of service employees (Etgar, 2008; Haumann et al., 2015; Ranjan & Read, 2016). Nowadays, most airlines rely on unassisted check-in (90%) or bag-drop systems (55%) (SITA, 2019) and 95% of US-consumers have experienced self-checkout kiosks in retailing (PYMNTS, 2018). Furthermore, due to the Covid-19 pandemic, products that require self-assembly by customers are among the fastest-growing e-commerce categories (Statista, 2020), with the market for ready-to-assemble furniture alone expected to grow by USD 17.5 billion from 2020–2024 (Business Wire, 2020). Driven by advances in digital technologies and artificial intelligence, the next big wave of coproduced services is emerging in the healthcare sector with fundamental changes for how medical services will be provided in the future (Mathews et al., 2020).

The widespread appeal of coproduction concepts builds on the belief that they enable firms to enhance production efficiency and reduce costs while offering various customer benefits at the same time (Detecon Consulting, 2014; Prahalad & Ramaswamy, 2000). In line with this win–win perspective, prior research on coproduction has identified various customer benefits such as accessibility, autonomy, and customization and emphasized how these benefits drive customer satisfaction and willingness to pay (e.g., Atakan et al., 2014; Dahl & Moreau, 2007; Norton et al., 2012; Troye & Supphellen, 2012; Zhu et al., 2007). However, some studies have also pointed out negative responses such as reduced satisfaction when coproduction concepts are perceived as effortful and time-consuming (Haumann et al., 2015; Stokburger-Sauer et al., 2016), do not lead to the desired outcome (Heidenreich et al., 2014), or lack alternative service options (Buell et al., 2010; Reinders et al., 2008).

In light of these mixed findings, recent reviews have questioned the general enthusiasm for coproduction and have called for a better understanding of a) when and why coproduction concepts yield positive or negative consumer responses and b) how these responses influence customer relationships over time (Dong & Sivakumar, 2017; Shin & Perdue, 2019). Although prior research on coproduction offers valuable cross-sectional insights, adopting a temporal perspective is necessary to address the fact that customer relationships are inherently dynamic and subject to change over time. Therefore, a pressing but unanswered question is how consumers process positive and negative perceptions of coproduction and how such ambivalent perceptions might differ in shaping the development of customer outcomes over time.

The present research makes a first attempt to address these questions by investigating how positive and negative perceptions of coproduction influence customer satisfaction and willingness to pay over time. Drawing from the multiple inference model of attribution (Reeder et al., 2004), we propose a conceptual framework that captures the ambiguity involved in customer perceptions of coproduction in a dual attribution process, in which customers hold positive (customer-serving) and negative (firm-serving) attributions simultaneously. While attributions of customer-serving motives reflect the positive belief that a firm offers coproduction to address its customers’ needs (e.g., convenience, accessibility, etc.), attributions of firm-serving motives reflect the negative belief that a firm offers coproduction to fulfill egoistic goals (e.g., cost cutting, service staff reduction). In a longitudinal field study (n_1_ = 12,662), a randomized experiment (n_2_ = 931), and a cross-sectional field study (n_3_ = 360), we investigate 1) how firm- and customer-serving motive attributions affect important customer outcomes and spending behavior, 2) how these effects develop over time, and 3) how firm decisions related to the degree of customer participation (in design and realization activities) and pricing shape customers’ attributions of firm motives.

Our results show that customer-serving motive attributions positively affect customers’ satisfaction and willingness to pay, whereas firm-serving motive attributions negatively affect both outcomes. Notably, these effects follow systematically different patterns over time: While the positive effects of customer-serving motive attributions decrease over time, the negative effects of firm-serving attributions are temporally much more stable. These effects also translate to customer spending behavior. With respect to the drivers of these processes, we show that more intense customer participation in the realization stage of the coproduction process increases firm-serving and decreases customer-serving motive attributions. Conversely, more freedom to customize the outcome in the design stage and higher monetary savings through coproduction strongly increase customer-serving motive attributions but have little effect on firm-serving motive attributions.

Hereby, the present research makes several theoretical contributions and extends previous research on coproduction in multiple ways (cf. Table 1). First, it advances the coproduction literature by providing novel insights into the temporal dynamics that guide short- and long-term effects of coproduction on customer relationship outcomes. While cross-sectional research has documented positive and negative customer responses to coproduction (for a review see Dong & Sivakumar, 2017), we apply a longitudinal perspective to examine how the effects of ambivalent attributions of a firm’s coproduction motives shape the development of customer outcomes over time. Specifically, we provide evidence for a temporal negativity bias according to which the influence of positive coproduction attributions on customer outcomes gradually decreases over time, while the influence of negative attributions is temporally much more stable. By identifying this temporal negativity bias, the present research challenges the assumption that the effects of coproduction will generally fade over time as customers become used to these offerings (e.g., Wang et al., 2013) and clarifies that a temporal decay only occurs for positive perceptions, while the effects of negative perceptions on customer outcomes are more enduring.

**Table 1:** Study 1: Overview of contribution to related literature

Study	Context	Data (sample size)	Characteristics of customer participation concept (IV)			Psychological mechanism (mediators)	Outcomes (DV)	Longitudinal perspective	Differences in temporal effects	Validation with customer spending data	Integration of positive and negative evaluations	Key findings
			Design stage	Realization stage	Monetary savings							
Atakan et al. (2014)	Craft products (e.g., travel mug, CD)	Lab experiments (N = 444)	✓	✓	–	Identification with product, affective commitment to product	Product evaluation	–	–	–	–	Consumer participation in the realization stage (physical production) enhances affective commitment to the product but does not result in identification with the product. Participation during the design stage (input-specification) enhances identification and affective commitment, which in turn enhances evaluation of the self-made product. Finally, engaging consumers in both the realization and design stages does not create value over and above the main effects created by a high level of participation in either stage.
Buechel and Janiszewski (2014)	Toys (e.g., bunny craft kit)	Lab experiments (N = 565)	✓	✓	–	Process feelings (infuse creativity, irritated)	Product evaluation	–	–	–	–	The effects of assembly effort on product evaluations depend on the integration of the customization and assembly processes. When both processes are segregated, more assembly effort leads to lower product evaluation. When they are integrated, more assembly effort leads to higher product evaluation.
Haumann et al. (2015)	Ready-to-assemble furniture,	Field experiment (n = 803), Lab experiment (n = 821) Scenario experiments (N = 4,783)	–	✓	–	–	CP process satisfaction	–	–	–	–	Intense coproduction processes negatively affect customer satisfaction with the process. This negative effect can be mitigated by employing communication strategies that either emphasize coproduction value (value-enhancing communication strategies) or highlight optional coproduction service supplements (intensity-reducing communication strategies).
Scherer et al. (2015)	Roadside assistant service	Longitudinal usage data (n = 5,467)	–	✓	–	–	Usage behavior	✓	–	–	–	Results show that the self-service ratio (usage of self-service option in relation to total service usage) directly impacts the likelihood that customers stop using the service in a U-shaped manner. This effect is mitigated across the study's timeframe.
Wang et al. (2013)	Grocery self-checkout	Longitudinal field study (n = 268)	–	✓	–	satisfaction, habit, self-efficacy	SST usage	✓	–	–	–	Customers’ continued use of an SST is initially largely rational driven (self-efficacy), then largely emotional driven (satisfaction), and, finally, habitually driven (habit). Habit fully mediates the impact of intentions on behavior.
Nijssen et al. (2016)	Grocery self-checkout	Field study (n = 110)	–	✓	–	Attributions of CP motives (cost, benefit)	Store evaluation	–	–	–	✓	Attributions mediate the impact of SST evaluation on store evaluation. Benefit attributions have a positive main effect on store evaluation, while the effect of cost attributions is not significant. However, there is an interaction between both motive attributions, such that benefit attributions only influence store evaluations when cost attributions are low.
Dong and Sivakumar (2018)	Grocery self-checkout	Scenario experiments (N = 783)	–	✓	–	Attributions of CP motives (customer-serving)	Customer satisfaction	–	–	–	–	Inferences of positive firm motives increase customer satisfaction and customers infer less positive firm motives for low-tier (vs. high-tier) brands offering coproduction. This brand-tier effect is moderated by customer autonomy and expectations towards the brand-tier.
Current study	Ready-to-assemble furniture	Longitudinal field study (n = 12,662, 6 waves, 32 weeks) Scenario experiment (n = 931) Field study (n = 360)	✓	✓	✓	Attributions of CP motives (firm-serving, customer-serving)	Customer satisfaction, willingness to pay more	✓	✓	✓	✓	Customers hold ambivalent attributions of a firm’s coproduction motives (firm- and customer serving) that have systematically different effects on key customer outcomes over time. Whereas the positive effects of customer-serving motive attributions decrease over time, the negative consequences of firm-serving attributions are much more stable. Customers’ attributions of firm motives are shaped by key characteristics of a coproduction concept (i.e., coproduction intensity, design freedom, and monetary savings).

IV = independent variable, DV = dependent variable, CP = coproduction, SST = self-service technology, DS = Design stage, RS = Realization stage, MS = monetary savings. Please note that this overview only features work that is related to the theme of the current study and is not meant to provide a complete overview of the literature on coproduction. For the latter, we refer the interested reader to review articles by Dong and Sivakumar (2017) and Shin and Perdue (2019)

Second, we advance the sparse knowledge on psychological mechanisms that explain how coproduction shapes customer relationships (Dong & Sivakumar, 2017), by proposing that these temporal developments can be explained by a dual motive attribution process. While inferred firm motives have been shown to play a relevant role in other business contexts, such as corporate social responsibility campaigns (e.g., Habel et al., 2016) and sport sponsorships (Woisetschläger et al., 2017), we show how inferred firm motives advance our theoretical understanding of how customers interpret and respond to ambivalent motive attributions over time. By shedding first light on the temporally diverging effects of customer- and firm-serving motive attributions on customer relationship outcomes, we further extend knowledge on the short- and long-term effects of inferred firm motives in general (Sipilä et al., 2020).

Third, the present study advances the integration of the largely isolated research streams on customer participation in the design and realization stage (Atakan et al., 2014), by proposing a mechanism that explains how consumers integrate their experiences in both stages. Our research adds the notion that a firm’s decisions related to the degree of customer participation in the design stage (i.e., design freedom) and the realization stage (i.e., coproduction intensity) serve as diagnostic cues for customers to infer firm- and customer-serving coproduction motives. Moreover, we also consider the role of pricing in coproduction (i.e., perceived monetary savings) and how it affects customers’ inferred coproduction motives. With this integrated perspective, our research helps to understand the different conclusions regarding the benefits of coproduction concepts between both literature streams (Dong & Sivakumar, 2017) and advances knowledge on when and why coproduction concepts have positive or negative consequences for customer relationships.

From a managerial perspective, the present research indicates that firms need to develop a greater awareness of the differential short- and long-term effects of coproduction concepts on customer relationships. We show that the current cross-sectional perspective on coproduction leads managers to underestimate potentially detrimental long-term consequences of ill-designed coproduction concepts. To overcome this bias, our research provides an understanding of the underlying mechanisms that explain differential temporal consequences of positive and negative coproduction perceptions and offers guidance on how firms need to integrate decisions on the degree of customer participation (in the design and realization stage) and product/service pricing to develop more successful coproduction concepts in the long run.

## Literature review on research on coproduction

### Customer coproduction in the design and realization stage

Customer coproduction refers to firm-initiated offerings in which customers actively participate in the production of the core good or service offering with no direct involvement by the focal firm or its employees (Dabholkar, 1996; Haumann et al., 2015; Meuter et al., 2000). This definition captures both coproduction concepts in which customers participate in the production of tangible outcomes (e.g., ready-to-assemble furniture, ready-to-cook meal kits) and intangible outcomes (e.g., self-service checkouts, self-service restaurants) (Haumann et al., 2015).^1^

Firms mainly offer customer participation in two distinct stages of the production process: the design and/or the realization stage (Atakan et al., 2014). Customer participation in the design stage implies that customers can make choices about product or service options to customize an outcome (e.g., design kitchen layout). Customer participation in the realization stage implies that customers invest effort and time to produce a tangible (e.g., furniture assembly, preparing a meal kit) or intangible outcome (e.g., self-checkout in retailing, online booking of a flight). Although some firms only offer customer participation in one of the two stages, many firms employ concepts in which customers can participate in both stages (e.g., planning and building a cabinet at IKEA, preparing a HelloFresh meal kit and add personal variations; Buechel & Janiszewski, 2014; Troye & Supphellen, 2012).

### Positive and negative customer responses to coproduction

Prior research has mainly highlighted the benefits of coproduction for customers and firms (Dong et al., 2015). Accordingly, engaging customers in coproduction has been shown to increase consumers’ perceived control over the production process and outcome (Bateson, 1985; Zhu et al., 2007), increase the accessibility and availability of products/services (Collier & Kimes, 2013), heighten consumers’ sense of self-competence (e.g., Dahl & Moreau, 2007; Mochon et al., 2012), and strengthen consumers’ emotional bond with the outcome (Atakan et al., 2014). Offering possibilities to customize an outcome can further increase preference fit (e.g., Franke & Piller, 2004), better allow consumers to express their identity (e.g., Buechel & Janiszewski, 2014; Troye & Supphellen, 2012) and develop stronger psychological ownership for the outcome (Wiecek et al., 2020).

However, initial evidence suggests that customers do not always experience benefits in coproduction and are even less satisfied when coproduction processes demand more effort and time (Haumann et al., 2015; Stokburger-Sauer et al., 2016), do not result in a desired outcome (Heidenreich et al., 2014), or when full-service options are lacking (Buell et al., 2010; Reinders et al., 2008). Moreover, advertising customer participation options can also make participation costs more salient and thereby decrease willingness to pay (Stadler-Blank & Bolton, 2019). Thus, taken together, prior research offers mixed results on the question whether coproduction yields positive or negative customer responses.

### Short- and long-term consequences of positive and negative coproduction perceptions

In light of the mixed customer responses to coproduction, a pressing question is how adopting such a concept shapes customer relationships over time (e.g., Shin & Perdue, 2019). While practitioners often view coproduction as an “instrument for generating customer loyalty” (Detecon Consulting, 2014, p. 7), there is yet little empirical evidence to support this claim.

To the best of our knowledge, only two prior studies have adopted a longitudinal perspective on coproduction. Wang et al. (2013) focus on how the intention and habit of using a self-checkout option in supermarkets influences continued usage of self-service and Scherer et al. (2015) investigate how the ratio of using a self-service technology (vs. a personal service option) affects ongoing usage of a roadside assistance service system. These studies provide valuable insights into how the adoption of a coproduction concept and how the frequency of using a self-service option affect ongoing usage behavior. However, they are not informative to understand the potentially differential effects of positive and negative customer perceptions to coproduction and how these might shape customer outcomes over time. Thus, the essential question of how positive and negative coproduction perceptions shape customer relationships over time remains yet unanswered; which has stimulated recent calls for more research on the short- and long-term relationship consequences of coproduction concepts (e.g., Dong & Sivakumar, 2017; Shin & Perdue, 2019).

The present research makes a first attempt to address these calls. We draw from the multiple inference model of attribution (Reeder et al., 2004) to provide an understanding of the underlying psychological mechanism that explains how customers cope with both positive and negative coproduction perceptions and how they integrate these to shape their responses to a focal firm. We complement this theory framework with insights from research on the negativity bias (e.g., Baumeister et al., 2001) to explain the temporal development of these effects. We introduce this theoretical framework in the following and then develop our hypotheses.

## Theoretical framework and hypotheses development

### The role of firm- and customer-serving motive attributions

Attribution theories explain how people make inferences about others’ underlying motives and character based on their observable behavior (Heider, 1958; Jones & Davis, 1965). Understanding attribution processes is important for predicting consumer behavior (Folkes, 1988), especially in situations in which firms modify traditional responsibilities in exchange relationships, such as when deciding to engage customers in coproduction processes (Bendapudi & Leone, 2003). This firm-initiated change is likely to trigger customers’ inference making about the firm’s underlying motives: For instance, customers might question why certain products demand more or less user assembly or why a firm offers a self-checkout instead of a regular cashier (Nijssen et al., 2016). The answers to these questions shape the inferences that customers make about the motives underlying a firm’s coproduction concept and, in turn, the valence of their attitudes and behavioral responses towards the firm.

The multiple inference model (MIM; Reeder et al., 2004) augments the basic principles of attribution theory by offering a framework that explains how people integrate multiple motive attributions. This theory holds that – in the same way that people’s actions may be guided by more than one motive at a time – people can simultaneously entertain multiple, plausibly rival hypotheses about the motives underlying others’ behavior (Ellen et al., 2006). There are two basic types of motive attributions: Self-serving motive attributions reflect a perceiver’s belief that a target’s behavior is driven by a motivation to realize selfish benefits, while other-serving motive attributions reflect beliefs that a target’s behavior is driven by a motivation to address others’ needs and wants (Batson, 1990). Notably, these two motive attributions are not opposite ends of a continuum, but independent dimensions that can exist simultaneously (Ellen et al., 2006; Fein, 1996).

**Fig. 1:**
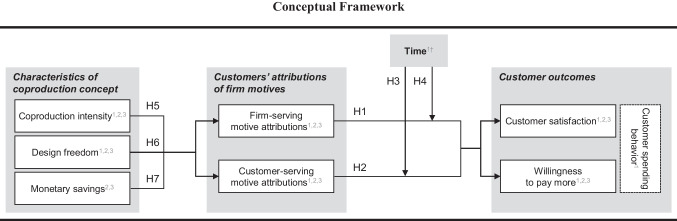
Conceptual framework. Notes: ^1^ Tested in Study 1. ^2^ Tested in Study 2. ^3^ Tested in Study 3. ^†^ We expect that the effects of firm- and customer-serving motive attributions on both outcomes differ over time. We analyze these different temporal patterns by estimating a dual-process, second-order latent growth model comprising six measurement waves (t_0-5_). The moderating influence of time is then assessed by comparing the effects of customer- and firm-serving motive attributions on the latent slope parameters of the customer satisfaction willingness to pay more trajectory (H3-4). Time-invariant controls: Product category involvement; coproduction experience; customer relationship length with the firm; propensity for DIY projects; coproduction situation (alone or with someone); gender; age; income. Time-varying controls: reception of positive word of mouth and/or news coverage about focal company (t_0-5_), reception of negative word of mouth and/or news coverage about focal company (t_0-5_), positive experience with focal firm (t_0-5_), negative experience with focal firm (t_0-5_), indicator of whether a customer moved within the last 6 weeks (t_0-5_)

Drawing on this theory, we define *firm-serving motive attributions* as a type of self-serving motive attribution that reflects customers’ beliefs that a firm offers coproduction to realize selfish goals such as cutting labor costs or increasing firm profits. In contrast, we define *customer-serving motive attributions* as a type of other-centered motive attribution that reflects customers’ beliefs that a firm offers a coproduction concept to satisfy its customers’ goals, e.g., in terms of a better preference fit or easier product accessibility. We conceptualize these motive attributions as distinct beliefs that can exist simultaneously and thus incorporate them as separate constructs in our conceptual model (see Fig. 1).

To gain insights into the short- and long-term relationship consequences of ambivalent attributions of coproduction motives, we investigate how the effects of firm- and customer-serving motive attributions on customer relationships are moderated by time, that is, how these effects develop over time. For customer relationship outcomes, we rely on *customer satisfaction*, as an outcome capturing customers’ global evaluation of a firm, and customers’ *willingness to pay more*, as an outcome that is more financially relevant (Homburg et al., 2005). We selected these outcomes because they are the most frequently studied outcomes in prior coproduction research (Dong & Sivakumar, 2017) and relate to different but important aspects of a customer relationship (Katsikeas et al., 2016). We further cross-validate these effects by assessing the impact of inferred firm motives on objective customer spending data.

If attributions of coproduction motives indeed influence customer relationship outcomes, it is important for managers to learn more about how customers make inferences about these motives. As each firm adopting a coproduction concept must decide on the degree of customer participation in the design and/or realization stages (Atakan et al., 2014) and the pricing of their coproduction concept (Stadler-Blank & Bolton, 2019), we propose that the outcomes of these decisions are diagnostic for customers to draw inferences about a firm’s coproduction motives. Therefore, we consider customer perceptions of three key characteristics of a coproduction concept as antecedents in our conceptual model: customer perceptions of 1) *coproduction intensity* (the effort and time customers required to create an outcome in the realization stage; Haumann et al., 2015), 2) *design freedom* (the number or quality of options available to customize the outcome in the design stage; Moreau et al., 2020), and 3) *monetary savings* (i.e., the amount customers can ‘save’ due to their own participation in coproduction).

In the following, we draw from the multiple inference model (Reeder et al., 2004) to develop hypotheses about differential short- and long-term effects of firm- and customer-serving motive attributions on customer outcomes. Further, we present our hypotheses on how the degree of customer participation in the design stage (design freedom) and the realization stage (coproduction intensity) as well as the firm’s price setting (monetary savings) affect customers’ inferences of a firm’s coproduction motives.

### Consequences of firm- and customer-serving motive attributions

Attributions of firm-serving motives reflect customers’ belief that a firm employs a coproduction concept to serve selfish goals such as cutting costs or achieving productivity gains. Although such economic goals are inherent to the objectives of for-profit firms and thus widely accepted among stakeholders (Ellen et al., 2006), they may trigger negative reactions when associated with opportunistic outsourcing of production activities to the customer. In the context of coproduction, attributions of firm-serving motives are thus likely to be perceived as non-reciprocal or even exploitive firm behaviors when customers have the impression that a firm internalizes their benefits instead of sharing them (e.g., by reducing prices). Following the MIM, attributions of self-serving motives should thus have a negative connotation and lead to less favorable responses to a target (Reeder et al., 2004). Hence, when customers believe that a firm engages them in coproduction in order to cut labor costs and improve profitability at their expense, customers will be less satisfied and reciprocally reduce their relationship investments by lowering their willingness to pay for the firm’s products and services. Thus, we expect that:

#### H1

Firm-serving motive attributions have a negative effect on a) customer satisfaction and b) willingness to pay more.

Attributions of customer-serving motives indicate a customer’s belief that the firm employs a coproduction concept in order to better satisfy its customers’ needs and wants. According to the MIM, such other-serving motive attributions are associated with positive traits of the respective target and are thus more likely to lead to favorable responses (Reeder et al., 2004). This is in line with the principle of reciprocity, which suggests that people respond more favorably to others who are believed to have good intentions towards them (Gouldner, 1960; Homans, 1958). In the context of customer attributions of coproduction motives, we thus expect that customers will respond more favorably when they believe that a firm offers coproduction due to a genuine motivation to better address customer needs (Dong & Sivakumar, 2018). Thus, in response, customers are more likely to be satisfied with the firm and reciprocate its investment in the relationship by being willing to pay higher prices. Therefore, we hypothesize that:

#### H2

Customer-serving motive attributions have a positive effect on a) customer satisfaction and b) willingness to pay more.

### Consequences of firm- and customer-serving motive attributions over time

In addition to the initial effects of coproduction motives on customers’ satisfaction and willingness to pay more, we offer first insights into the development of these effects over time. To develop hypotheses on the consequences of firm motive attributions over time we integrate the theoretical framework of the MIM with research on temporal effects of the negativity bias (Baumeister et al., 2001). The negativity bias suggests that “negative information weighs more heavily on the brain” (Ito et al., 1998, p. 887), because negative information is often processed more thoroughly than positive information and thus leaves a more indelible trace in the memory (Baumeister et al., 2001; Rozin & Royzman, 2001). This negativity bias implies a systematic difference between the storage rates of negative and positive information, such that people have greater memory for negative than for positive information of others (Pratto & John, 1991) and experience more enduring emotional reactions resulting from negative compared to positive information (Ikegami, 1993). For example, studies show that people have a better memory for faces of cheaters (i.e., non-reciprocators) than for cooperators (Bell & Buchner, 2009) because “memory regarding cheaters is more persistent against extinction than memory regarding cooperators” (Suzuki et al., 2013, p. 1902). In sum, research on the negativity bias suggests that negative information has more long-lasting consequences than positive information.

We integrate these insights on the temporal effects of the negativity bias with the theoretical notions of the MIM to suggest that the consequences of self- and other-serving motive attributions follow different patterns over time. While both motive attributions should generally decay over time, this theoretical rationale suggests that self-serving (negative) motive attributions decay at a slower rate and have more sustained consequences than other-serving (positive) motive attributions. In the context of our study, we thus expect that the negative effects of attributions of self-serving firm motives decay at a lower rate than the positive effects of customer-serving motive attributions. Therefore, we predict that:

#### H3

The positive effects of customer-serving motive attributions on a) customer satisfaction and b) willingness to pay more decrease over time.

#### H4

The negative effects of firm-serving motive attributions on a) customer satisfaction and b) willingness to pay more are more persistent over time than the positive effects of customer-serving motive attributions.

### Effects of coproduction characteristics on inferred coproduction motives

In the following, we turn to the antecedents of customers’ attributions of coproduction motives. A key decision in a firm’s adoption of a coproduction concept relates to the extent to which customers are engaged in the realization stage, which translates to the perceived effort and time that customers have to invest in the production process. While firms are motivated to cut costs by substituting employee with customer labor (Bendapudi & Leone, 2003), outsourcing these processes implies higher non-monetary costs and thus a less favorable outcome/input ratio for customers (Haumann et al., 2015). If customers are not compensated for this (e.g., through lower prices or more design freedom), they may feel that the company is only involving them in order to have less effort itself (Lengnick-Hall, 1996). Accordingly, we expect that higher levels of coproduction intensity increase customers’ attributions of firm-serving coproduction motives.

Analogously, higher levels of perceived intensity also decrease perceptions that a firm employs a coproduction concept to serve its customers’ needs. As customers prefer convenient and time-efficient coproduction processes (Collier & Kimes, 2013), firms that offer a concept that falls short on these parameters may seem less responsive to their customers’ needs. In this sense, higher levels of coproduction intensity are likely to lead consumers to attribute less customer-serving motives to the firm. Following the preceding arguments, we thus hypothesize:

#### H5

Perceived coproduction intensity is a) positively associated with firm-serving motive attributions and b) negatively associated with customer-serving motive attributions.

A second diagnostic characteristic refers to the decision of a firm to offer opportunities to alter the design or specification of a coproduced outcome (Troye & Supphellen, 2012), which is reflected in customers’ perceived design freedom in the process. A firm’s decision to offer more design freedom for customers reflects a desire to fulfill customer needs for autonomy (Dahl & Moreau, 2007) or a better preference fit (Franke & Piller, 2004), we expect that higher levels of design freedom will lead to stronger inferences of customer-serving firm motives.

Offering more freedom for customers to change and adapt the design or specification of a coproduced outcome demands more effort for firms, as they need to offer a greater variety of product attributes (Kahn, 1998) or implement customization kits (Randall et al., 2007). Hence, greater design freedom may serve as a signal for customers, indicating that a firm is willing to invest in enhanced customer benefits. Experiencing more design freedom may thus also trigger customers’ beliefs that a firm does not prioritize self-serving benefits, but is willing to go the extra mile to deliver superior products or services to its customers. Thus, we propose:

#### H6

Perceived design freedom is a) negatively associated with firm-serving motive attributions and b) positively associated with customer-serving motive attributions.

A third diagnostic characteristic refers to the price setting for coproduced products and services, which reflects customers’ perceived monetary savings through coproduction; i.e., the extent of perceived savings compared to a full-serviced option or a comparable product from a competitor that requires no customer participation. As engaging customers in coproduction enables firms to realize cost savings (Bendapudi & Leone, 2003), customers might expect firms to market the offering at a lower monetary price to compensate customers for their participation in the production process. As the extent to which a firm’s prices allow customers to save money is diagnostic for their attributions of underlying firm intent (Habel et al., 2016), we expect that higher monetary savings increase customers’ attributions of customer-serving coproduction motives and decrease their attributions of firm-serving motives. Thus, we hypothesize that:

#### H7

Perceived monetary savings through coproduction are a) negatively associated with firm-serving motive attributions and b) positively associated with customer-serving motive attributions.

## Overview of studies

We conducted two qualitative pre-studies and three quantitative main studies to test our hypotheses. The two pre-studies offer initial insights into the prevalence of firm- and customer-serving motive attributions in different coproduction contexts. Study 1 is a longitudinal field study and our focal study to investigate the development of the effects of firm- and customer-serving motive attributions on customer satisfaction and willingness to pay over time (H1-H4). As it builds on actual customer-company relationships, it also allows us to validate and quantify the downstream consequences of the proposed effects with objective data on customer spending behavior. In Study 1, we also offer an initial test of the inference model based on the degree of customer participation in the design and realization stage (H5-H6). Study 2 is an experiment in which we manipulate the extent of coproduction intensity, design freedom, and monetary savings to offer causal evidence on their role in shaping customers’ inferences of a firm’s coproduction motives (H5-H7). Study 2 also provides additional tests for the effects of inferred firm motives on customer outcomes (H1-H2). Study 3 is a field study among participants from different firms, in which we validate our findings on the effects of inferred firm motives on customer outcomes (H1-H2) and the relationships of coproduction characteristics with inferred firm motives (H5-H7).

## Qualitative pre-studies

**Fig. 2:**
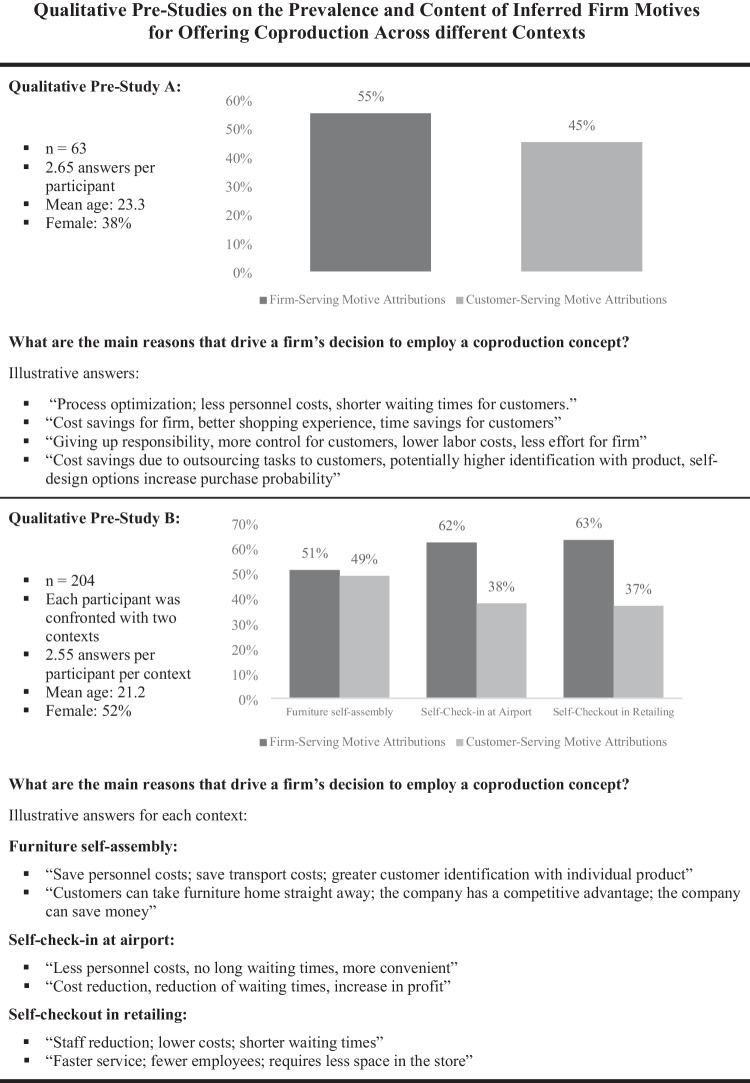
Qualitative pre-studies on the prevalence and content of inferred firm motives for offering coproduction across different contexts

To provide first systematic evidence on the prevalence of inferred firm motives across different coproduction contexts, we conducted two qualitative pre-studies. We recruited business administration students from two different public universities as participants and asked them what they thought why companies offer coproduction concepts (see Web Appendix A for more details on the procedure). While we asked one sample about their general perception (n_1_ = 63, mean age 23.3, female 38%), the other sample referred to the most common coproduction contexts, i.e., ready-to-assemble furniture, self-checkout in retailing, and self-check in at airports (n_2_ = 204, mean age 21.2, female 52%). Two independent coders who were blind to our hypotheses categorized the responses into reasons that either reflect firm- and/or customer-serving motives. In sum, the results show that respondents believe that firms are driven by both, firm- and customer-serving objectives. A content analysis of the answers further revealed that firm-serving motive attributions are mainly associated with a firm’s striving for cost savings, whereas customer-serving motive attributions are mostly associated with customer benefits, such as money and time savings, convenience, or autonomy. Overall, the frequency of firm- and customer-serving motives was balanced in the furniture assembly context (51% vs. 49%), while respondents seem to attribute more firm-serving motives for self-check-in systems at airports (62% vs. 38%) and self-checkout systems in retailing (63% vs. 37%) (see Fig. 2).

The pre-studies’ results clearly indicate that consumers attribute both firm-and customer-serving firm motives for offering a coproduction concept. Building on these qualitative insights, the following quantitative studies examine the role of both motives in explaining when and why coproduction has positive or negative consequences for customer-firm relationships over time.

## Study 1: Longitudinal field study

The goal of Study 1 is to test our conceptual model in a longitudinal field study involving actual customers of a firm that offers customer participation in the design and the realization stage.^2^ We chose ready-to-assemble furniture as the context for our study for multiple reasons. First, this context allows us to study customer participation in design and production activities within a single setting (Atakan et al., 2014). Second, furniture assembly is one of the most prototypical coproduction contexts (Bendapudi & Leone, 2003; Norton et al., 2012) and thereby facilitates the integration and comparison of our study’s findings within the body of prior work. Third, our qualitative study indicates that this context offers the most conservative test of our hypotheses on inferred firm motives, as the share of firm-serving motive attributions is a) substantially lower than in other coproduction contexts and b) on an equal level with the number of customer-serving motive attributions (see Fig. 2).

### Method

#### Data collection and procedure

We collaborated with a multinational firm that offers ready-to-assemble furniture to collect data on actual customer relationships. The firm provided us with a dataset of customers including email addresses, basic demographics (gender), and information on individual customer spending behavior (i.e., sum of spending per study wave). We used the email addresses provided by the firm to set up a longitudinal panel study, in which we approached customers in six measurement waves (approximately every 6 weeks) over a data collection period of 32 weeks. The invitation stated that the study was part of a research collaboration between the focal firm and a large public university. We incentivized participation in each wave with a lottery for several prizes (shopping vouchers, tablets, etc.) and incentivized participation across all study waves with an additional lottery only among the respondent that met this criterion.

Upon entering the survey, we briefed participants about the collaboration project with the firm and the general data protection guidelines. Participants were then introduced to the topic of the study and responded to the measures for our key constructs, covariates, and demographics. While the front-end variables of the model were only captured in the first measurement occasion, we re-assessed the variables relevant for the temporal analysis in all subsequent study waves.

#### Response rate and sample demographics

Of the 74,254 customers that were invited to participate in the study, we received 12,794 responses. We excluded 132 respondents who indicated that they had no experience with the coproduction concept of the firm, thus resulting in an overall sample of 12,662 respondents that could be included in the model estimation process. The resulting response rate of 17.05% is comparable to similar longitudinal consumer studies (e.g., Ahearne et al., 2010; Rindfleisch et al., 2009). We conducted several additional analyses to rule out issues with non-response bias and assure that the demographic and spending profile of our sample (mean age 37.85, female 77.7%) is representative of the firm’s customer profiles (see Web Appendix B).

#### Measures

Appendix 1 provides a detailed overview of all measurement items used in this study. Attributions of firm- and customer-serving coproduction motives were each measured with a three-item scale adopted from previous research (Ellen et al., 2006; Vlachos et al., 2009), which captured the extent to which customers think that the firm offers a self-assembly concept to pursue firm-serving goals (customer-serving goals). To measure customer satisfaction, we used a three-item scale from Homburg et al. (2006) that captures overall satisfaction with the firm. For customers’ willingness to pay more, we used a three-item scale from Srinivasan et al. (2002) which captures customers’ willingness to pay a price premium at the focal firm compared to the competition. The items for the outcome measures were kept constant across the six measurement waves.

For customers’ perception of coproduction characteristics, we measured design freedom by adapting three items from Spreitzer (1995) that capture the extent to which customers perceive to have influence on the specification and design of the coproduced outcome. We measure coproduction intensity with five items that capture the extent to which customers perceive the coproduction activities at the firm to be demanding in terms of effort and time investments (Franke & Schreier, 2010; Haumann et al., 2015).

To assess the robustness of the proposed relationships and rule out potentially extraneous influences, we included several time-invariant controls in the empirical analyses (customers’ propensity for DIY projects, prior coproduction experience, relationship length with the firm, product category involvement, the coproduction situation [whether the furniture was assembled for oneself or for another person], gender, age, and income), which might be relevant for the development of inferred coproduction motives and customer outcomes. We further include several time-varying covariates in the model (Bollen & Curran, 2006) that we measured in each wave (t = 0 – 5), i.e., whether customers moved, whether they received positive or negative word of mouth and/or noticed positive or negative press coverage about the focal company, whether they had a positive or negative experience with the focal company since the last study wave.

#### Psychometric properties

Results of a confirmatory factor analysis show that the global measurement model fits the data well (CFI = 0.95; TLI = 0.92; RMSEA = 0.02; SRMR = 0.02). Web Appendix C shows the correlations and psychometric properties of our key measures. All measurement scales exceed values of 0.7 for coefficient alpha and thereby indicate sufficient internal consistency reliability of our construct operationalization (Nunnally & Bernstein, 1994). Moreover, composite reliability (CR) and average variance extracted (AVE) exceeded the recommended thresholds for all latent variables, providing further support for convergent validity (Bagozzi & Yi, 1988). To assess discriminant validity between different constructs for all measurement occasions, we used the criterion proposed by Fornell and Larcker (1981). This criterion suggests that discriminant validity is established when the AVE exceeds the squared correlations between all pairs of constructs. All constructs met this requirement.

#### Measurement invariance across time points

As we collected longitudinal data with constructs measured at multiple points in time, it is necessary to assess the longitudinal validity of these constructs by testing for measurement invariance (Ployhart & Vandenberg, 2010). In general, measurement invariance in longitudinal data sets is supported when configural and at least partial metric and scalar invariance is established (Vandenberg & Lance, 2000). To test for measurement invariance, we conducted a series of CFA nested model comparisons (Chan, 1998). The results support full configural, metric, and scalar invariance for all longitudinally measured constructs, thus providing support for the appropriateness of the data for longitudinal modeling.

#### Common method bias

Given our study design and modeling approach, it is unlikely that our findings are driven by common method variance for multiple conceptual reasons. In particular, we employed a longitudinal survey design (Podsakoff 2003), validate our findings with data from another source (here: objective firm data on customer spending), and find support for multiple theoretically plausible interaction effects (Siemsen et al., 2010). To further assure that common method variance is not a threat to the results and conclusions of the study, we additionally conducted multiple statistical tests. Specifically, we employed a confirmatory factor analytical approach to Harman’s single factor test (Griffith & Lusch, 2007), the marker-variable approach developed by Lindell and Whitney (2001), and the unmeasured latent method factor approach proposed by Podsakoff et al. (2003). Finally, we also evaluated an alternative latent growth model (with a lag 1 between the predictor variables and the variables of the latent growth trajectories). Results of all additional tests indicate that common method bias is not a threat to the results and conclusions of this study (see Web Appendix D).

#### Analytical procedure

We test our hypotheses using a latent growth model (Bollen & Curran, 2006) as this approach enables us to analyze longitudinal data at the individual customer level (Lance et al., 2000; Palmatier et al., 2013) and thereby test how the effects of inferred firm motives on customer outcomes develop over time. Conceptually, a latent growth model comprises two stages that are estimated simultaneously (Lance et al., 2000). In the first stage, a growth trajectory described by an intercept and a slope (in the case of a linear growth model) is fitted to the repeated measures (Duncan & Duncan, 2004). In the present model, we fit two growth trajectories for customers’ satisfaction and willingness to pay more spanning the observation period of 32 weeks. These trajectories are described by two latent intercept factors, reflecting the initial level of customer satisfaction and willingness to pay more, and two latent slope factors, indicating the change of these customer outcomes over time.^3^

In the second stage, predictors of the latent intercept and slope factors that explain differences in the individual growth trajectories are added to the model (Duncan & Duncan, 2004). In the present model, we included firm- and customer-serving motive attributions as predictors of the latent intercept and slope factors in the model to analyze their consequences for customer satisfaction and willingness to pay more over time. The initial effects of attributions of coproduction motives on both outcomes are reflected in the intercept coefficients, which are the basis for testing H1-2. The slope coefficients describe the development of these effects over time and are thus the basis for testing H3-4. Furthermore, coproduction design freedom and intensity are included as predictors of customers’ attributions of coproduction motives to test H5-6. Finally, we incorporate the previously discussed set of time-invariant and -varying covariates in the model estimation process. For additional details on the model specifications, see Web Appendix E. We used Mplus 8.4 for the analysis and employed a maximum likelihood estimator with robust standard errors (Muthén and Muthén 1998–2018).

### Results

#### Effects of inferred firm motives on customer outcomes

Before diving into the hypotheses testing, it is worth noting that we find no correlation between firm- and customer-serving motive attributions (r = –0.01, *n.s.*; see Web Appendix C. This finding offers support for our theoretical argument that these motive attributions are not opposite sides of the same coin, but two independent perceptual dimensions that customers can hold simultaneously.

Generally, the model fits the data well (CFI = 0.95, TLI = 0.94, RMSEA = 0.02, SRMR = 0.04). Results appear in Table 2. To test H1 and H2, we assessed the effects of inferred firm motives on the latent intercepts of customer satisfaction and willingness to pay more in the main effects model. In support of H1, we find that firm-serving motive attributions have a significant negative effect on the intercept of customer satisfaction (H1a: β_11_ = –0.042, *p* < 0.01) and willingness to pay more (H1b: β_13_ = –0.156, *p* < 0.01). We also find support for H2, as customer-serving motive attributions have a significant positive effect on the latent intercept factor of customer satisfaction (H2a: β_21_ = 0.387, *p* < 0.01) and willingness to pay more (H2b: β_23_ = 0.301, *p* < 0.01).

**Table 2:** Study 1: Core results of dual-process latent growth analyses

	Main Effects Model							
	DV = Customer Satisfaction				DV = Willingness to Pay More			
	Intercept β_i1_	(S.E.)	Slope β_i2_	(S.E.)	Intercept β_i3 _	(S.E.)	Slope β_i4_	(S.E.)
Influence of inferred CP Motives								
Firm-serving CP motives (β_1j_)	–.042***	(.006)	–.015	(.012)	–.156***	(.013)	.009	(.021)
Customer-serving CP motives (β_2j_)	.387***	(.013)	–.126***	(.021)	.301***	(.019)	–.054*	(.032)
Difference between CP Motive Effects								
Firm-serving CP motives – Customer-serving CP motives (|β_1j_| – |β_2j_|)			–.111***	(.024)			–.045	(.036)
Control Variables								
Product category involvement (β_3j_)	.105***	(.014)	.024	(.022)	.084***	(.022)	–.027	(.033)
CP experience (β_4j_)	.027***	(.007)	–.0001	(.013)	.020	(.013)	–.011	(.021)
CP design freedom (β_5j_)	.157***	(.013)	.018	(.020)	.164***	(.022)	.039	(.034)
CP intensity (β_6j_)	–.109***	(.009)	.011	(.015)	–.073***	(.016)	.021	(.026)
Customer relationship length (β_7j_)	.002	(.003)	–.002	(.005)	–.001	(.006)	.014	(.009)
DIY propensity (β_8j_)	–.035***	(.009)	.001	(.014)	.020	(.015)	.034	(.021)
CP situation (β_9j_)	.011	(.017)	–.020	(.030)	.021	(.032	–.028	(.052)
Gender (β_10j_)	–.055***	(.020)	.027	(.034)	.144***	(.036)	.006	(.059)
Age (β_11j_)	.003***	(.001)	–.001	(.001)	–.007***	(.002)	–.003	(.002)
Income (β_12j_)	–.020***	(.005)	.009	(.008)	–.008	(.008)	–.008	(.014)
	DV = Firm-Serving CP Motives				DV = Customer-Serving CP Motives			
	γ_i1_		(S.E.)		γ_i2_		(S.E.)	
Influence of CP Characteristics								
CP design freedom (γ_1j_)	–.089***		(.021)		.310***		(.018)	
CP intensity (γ_2j_)	.208***		(.014)		–.180***		(.012)	
Control Variables								
Product category involvement (γ_3j_)	–.006		(.024)		.089***		(.018)	
CP experience (γ_4j_)	.122***		(.013)		.069***		(.010)	
Customer relationship length (γ_5j_)	.014**		(.006)		–.004		(.004)	
DIY propensity (γ_6j_)	–.001		(.016)		.036***		(.012)	
CP situation (γ_7j_)	.055*		(.031)		–.041*		(.023)	
Gender (γ_8j_)	.226***		(.036)		–.252***		(.029)	
Age (γ_9j_)	–.003**		(.002)		.009***		(.001)	
Income (γ_10j_)	.041***		(.009)		.005		(.007)	

n = 12,662; *p < .1; **p < .05; ***p < .01 (two-tailed tests). Estimates show unstandardized coefficients; S.E. = Standard error; CP = Coproduction. Influences of additional time-varying control variables not presented for ease of interpretation. Additionally included time-varying control variables: reception of positive word of mouth and/or news coverage about focal company (t_0-5_), reception of negative word of mouth and/or news coverage about focal company (t_0-5_), positive experience with focal firm (t_0-5_), negative experience with focal firm (t_0-5_), indicator of whether a customer moved within the last 6 weeks (t_0-5_). Standard errors of differences in effect sizes are based on multivariate delta method (e.g., Bishop et al., 1975)

#### Development of effects of inferred firm motives on customer outcomes over time

To test H3 and H4, we analyzed and compared the effects of inferred coproduction motives on the latent slope factors of both customer outcomes (see Table 2 and Web Appendix F for a visualization of these effects). Results of the latent growth model support H3 by showing that customer-serving motive attributions have a significant negative effect on the latent slope of customer satisfaction (H3a: β_22_ = –0.126, *p* < 0.01) and a marginally significant negative effect on the latent slope of willingness to pay more (H3b: β_24_ = –0.054, *p* < 0.10, two-tailed). In line with H3, these results indicate that the positive effects of customer-serving motive attributions on both outcomes diminish over time.

H4 further suggests that the negative effects of firm-serving motive attributions are temporally more persistent than the positive effects of customer-serving motive attributions. In support of H4, results in Table 2 show that the effects of firm-serving motive attributions on the slope factor of customer satisfaction (H4a: β_12_ = –0.015, *n.s.*) and willingness to pay more (H4b: β_14_ = 0.009, *n.s.*) are both not significant, indicating that the negative effects of firm-serving motives remain unchanged and thus more stable over time. We draw additional support for H4 from a comparison of the effects of firm- and customer-serving motive attributions on the slope factor of each outcome. This comparison offers support for H4a by showing that the effect of customer-serving motive attributions on the latent slope factor of customer satisfaction is significantly stronger compared to the effect of firm-serving motives (H4a: ∆ (|β_12_ | – |β_22_ |) = –0.111, *p* < 0.01).

We observe the same pattern of results when comparing the effects of customer- and firm serving motive attributions on the latent slope factor of willingness to pay more, but find no statistically significant difference between both coefficients (H4b: ∆ (|β_14_ | – |β_24_|) = –0.045, *n.s.*). We conducted an additional analysis to offer insights into a potentially more complex nature of the dynamic relationship between inferred coproduction motives and willingness to pay more. We draw from research on individual difference factors that affect consumers’ willingness to pay (Koschate-Fischer et al., 2012) to explore the role of product category involvement as a potential contingency factor of the temporal dynamics in the effects of inferred firm motives on willingness to pay more. Results of this additional analysis (see Web Appendix G) show support for H4b for low (vs. high) involved customers. For low involved customers, the positive effect of customer-serving motive attributions significantly diminishes over time (β_ωL2_ = –0.100, *p* < 0.01), while the effect of firm-serving motive attribution does not decrease significantly (β_ωL1_ = –0.017, *n.s.*). The difference between these parameters is marginally significant (∆ (|β_ωL1_ | – |β _ωL2_ |) = –0.083, *p* < 0.10, two-tailed) and thereby indicates that the temporal dynamics proposed in H4b are especially pronounced for customers with a low level of involvement in the product category.

#### Effects of coproduction characteristics on inferred firm motives

To test H5 and H6, we assessed the effects of coproduction design freedom and intensity on firm- and customer-serving motive attributions (see Table 2). In support of H5, we find that higher coproduction intensity significantly increases firm-serving motive attributions (H5a: γ_21_ = 0.208, *p* < 0.01) and decreases customer-serving motive attributions (H5b: γ_22_ = –0.180, *p* < 0.01). We also find support for H6, as higher design freedom in coproduction decreases firm-serving (H6a: γ_11_ = –0.089, *p* < 0.01) and increases customer-serving motive attributions (H6b: γ_12_ = 0.310, *p* < 0.01). Thus, these coproduction characteristics serve as counteracting cues that customers rely on to make inferences about a firm’s underlying motivation to offer a coproduction concept.

### Additional analyses and robustness checks

#### Dominance of ambivalent coproduction motive attributions

To gain more nuanced insights into the joint effects of both motive attributions on customer satisfaction and willingness to pay, we relied on an established approach (see Vosgerau et al., 2008; Alavi et al., 2016) to examine the consequences of one motive attribution being more dominant than the other. To this end, we estimated a model incorporating the extent of dominance of one motive attribution over the other (operationalized as the absolute difference between both), the direction of dominance (dummy variable: 0 = ambivalent motive attribution; -1 = customer-serving motive attributions dominant; 1 = firm-serving motive attributions dominant), and the interaction effect between the extent and the direction of dominance.

Results of this model appear in Web Appendix H. They show a significant negative interaction between the extent and the direction of dominance on both intercept parameters (${\beta }_{31}=-.155, p<.01$, ${\beta }_{33}=-.174, p<.01)$ and non-significant effects on the slope parameters of the customer satisfaction and the willingness to pay more trajectory $({\beta }_{32}=.009, n.s.$, ${\beta }_{34}=-.001, n.s.$). The simple slopes provide further insights into the nature of the significant interaction effects. Specifically, the simple slopes show that a dominance of firm-serving motive attributions has negative effects (${\omega }_{11}=-.230, p<.01$, ${\omega }_{13}=-.279, p<.01$) whereas a dominance of customer-serving motives has positive effects on both outcomes compared to situations of equally strong held attributions $({\omega }_{21}=.080, p<.01$, ${\omega }_{23}=.070, p<.01$). Appendix 2, panel 1 and 2, illustrate these effects from weak (1) to strong (6) dominance.

#### Mediation analysis

We conducted a mediation analysis to test whether inferred firm motives mediate the relationship between coproduction characteristics and customer outcomes. Analyses of the indirect effects reveal that both firm- and customer-serving motive attributions mediate the effects of coproduction design freedom and intensity on the intercept and slope factors of customer satisfaction and willingness to pay more (see Web Appendix I). We further find that customer-serving motive attributions mediate the relationship between both coproduction characteristics and the slope factors of customers’ satisfaction and willingness to pay more.^4^ These findings further support the differential roles of firm- and customer-serving motive attributions as underlying mechanisms in guiding customers’ responses to coproduction over time.

#### Respondent attrition

Respondent attrition is a common issue in longitudinal research settings (e.g., Palmatier et al., 2007). As we incentivized participation in all study waves with an extra lottery, we observed a comparably small average respondent attrition rate, i.e., 16.67% per wave. To rule out potential biases that may result from respondent attrition, we estimated a model that explicitly accounts for respondent attrition (Enders, 2011). In line with methodological recommendations, we employed a pattern-mixture dropout modeling approach in which the latent intercept and slope factors of the growth model may vary as a function of a set of dummy variables that reflect the potential dropout occasion (Little, 1995; Muthén et al., 2011). This approach allows us to test whether respondent attrition at a specific measurement occasion affects the development of the growth trajectories and provides parameter estimates for the latent growth model that are corrected for a potential bias due to respondent attrition (Enders, 2011). Results of the pattern-mixture models show that respondent attrition does not substantially affect the results and hypotheses tests (see Web Appendix J) and thereby provide additional evidence for the robustness of our findings and conclusions.

#### Additional robustness checks

To further strengthen the robustness of our findings we conducted multiple additional robustness checks. Specifically, we ran additional analyses to investigate whether customers' gender, their coproduction experiences during our study, or the time since their last coproduction affect our findings. We discuss these robustness checks in greater detail in Web Appendix K. Results of the analyses underline the robustness of our findings.

#### Validation with objective data on customer spending

To validate our findings with objective data and to gain insights into the downstream consequences of inferred firm motives, we estimated an additional autoregressive model including firm data on customer spending (a detailed description of the model and its results appear in Web Appendix L). The results of this model show highly significant effects of customer satisfaction (β_2, CS_ = 3.293, *p* < 0.05) and willingness to pay more on customer spending (β_4, WTPM_ = 2.138, *p* < 0.01). Furthermore, we find significant total indirect effects of firm- and customer-serving motive attributions on customer spending via customer outcomes (β_IE1, Firm-serving_ = –0.731, *p* < 0.01; β_IE2, Customer-serving_ = 2.547, *p* < 0.01). Finally, we also find significant total indirect effects of coproduction design freedom and intensity on customer spending via both motive attributions and customer outcomes (β_IE3, DesignFreedom_ = 1.639, *p* < 0.01; β_IE4, CoproductionIntensity_ = –1.073, *p* < 0.01).^5^

### Discussion

Study 1 offers support for our conceptual framework in a field setting involving actual customer relationships. The unique longitudinal data set particularly enabled us to show that firm- and customer-serving motive attributions have diverging effects on customer satisfaction and willingness to pay more (H1-2) which systematically follow different temporal patterns and thereby differ in their short- and long-term impact on both outcomes (H3-4). We validated these effects with objective data on customer spending behavior to offer additional insights into the downstream consequences of inferred firm motives. Finally, the study provides first insights into how coproduction design freedom and intensity can serve as relevant cues for consumers to draw inferences about a firm’s coproduction motives (H5-6). To overcome the limitation that we cannot manipulate the characteristics that customers use as cues to draw inferences about a firm’s coproduction motives, we complement these insights with an experimental study.

## Study 2: Experimental study

The aim of Study 2 is to provide further evidence of the robustness of our proposed effects, particularly regarding the influence of coproduction characteristics on inferred firm motives. Besides coproduction intensity and design freedom, we also consider monetary savings to test H7. We used a randomized experiment with a 2 (intensity: low vs. high) × 2 (design freedom: low vs. high) × 2 (monetary savings: low vs. high) between-subjects design and situate the study in the context of ready-to-assemble furniture to offer a better comparison with Study 1.

### Method

#### Data collection and sample

We administered the study using an online questionnaire and used attention checks to ensure that participants have read and understood the manipulations (Hauser & Schwarz, 2016). The total sample comprises 931 undergraduate business students at a large public university (mean age 21.2, female 52%) who participated in return for extra credit.

#### Procedure

In line with prior studies on coproduction (e.g., Bendapudi & Leone, 2003), we manipulated the characteristics of the coproduction concept by using immersive scenarios. In each scenario, participants took on the role of a customer experiencing a coproduction process at a new furniture retailer. We manipulated the degree of coproduction intensity by altering the number of production steps necessary to complete a product (low = 10 steps; high = 50 steps) and the degree of design freedom by changing the number of available options to customize a product (low = 2 options; high = 216 options). The degree of monetary savings was manipulated by varying the price of the firm’s assembly service as a percentage of the products’ purchase price (low = 10%; high = 50%). The specific values for the manipulations were based on a screening of real market options and a qualitative pretest in a focus group.

#### Measures and psychometric properties

After reading the scenario, participants indicated their beliefs about the firm’s firm- and customer-serving motive attributions and indicated their satisfaction and willingness to pay. In the following, participants answered the same individual-level control variables as used in the field study, responded to attention and manipulation checks, and provided demographic information. We relied on the same measures as used in Study 1 (see Appendix 1). Results of a confirmatory factor analysis show that the global measurement model fits the data well (CFI = 0.98; TLI = 0.98; RMSEA = 0.03; SRMR = 0.03) and that all criteria for reliability and validity are supported (Bagozzi & Yi, 1988).

We also asked participants how realistic the described situation was (1 = “very unrealistic” to 7 = “very realistic”) and how well they could immerse in the scenario (1 = “not at all” to 7 = “totally”). Ratings of realism (mean = 5.58, *p* < 0.05) and immersion (mean = 5.70, *p* < 0.05) were both significantly above the scale mean. Finally, we openly asked whether participants had any idea about the study’s goal. None of the participants identified the goal.

#### Analytical approach

We relied on a structural equation modeling approach to analyze the data, as this approach enables us to estimate our model simultaneously and consider potential measurement errors of measurement scales (Kline, 2015).

### Results

#### Manipulation checks

As compared to the low-conditions, participants in the high-conditions perceived a higher degree of design freedom (M_low_ = 2.12; M_high_ = 5.98; F(1, 929) = 3350.12; *p* < 0.01), higher coproduction intensity (M_low_ = 2.19; M_high_ = 6.36; F(1, 929) = 5047.61; *p* < 0.01), and higher coproduction savings (M_low_ = 3.86; M_high_ = 5.17; F(1, 929) = 256.94; *p* < 0.01). No interactions reached significance. Thus, all manipulations worked as intended.

#### Effects of inferred coproduction motives on customer outcomes

In line with H1, we find negative effects of firm-serving motive attributions on willingness to pay more (H1b: β_12_ = –0.195, *p*<0.01) and customer satisfaction (H1a: β_11_ = –0.022, *n.s.*) (see Table 3). As the latter, however, does not reach statistical significance, the results only offer formal support for H1b but not H1a. Nevertheless, comparing these results with those of Study 1 reveals an unexpected but common pattern, as it seems that firm-serving motive attributions harm willingness to pay more than customers’ satisfaction. Regarding the consequences of attributions of customer-serving coproduction motives, we find significantly positive effects on both customer satisfaction (H2a: β_21_ = 0.768, *p* < 0.01) and willingness to pay more (H2b: β_22_ = 0.327, *p* < 0.01) that support H2.

**Table 3:** Study 2: Results of experimental study

	DV = Customer satisfaction		DV = Willingness to pay more	
	β_i1_	(S.E.)	β_i2_	(S.E.)
Influence of CP motives				
Firm-serving CP motives (β_1j_)	–.022	(.053)	–.195***	(.047)
Customer-serving CP motives (β_2j_)	.768***	(.051)	.327***	(.034)
Control Variables				
Product category involvement (β_3j_)	.006	(.036)	.036	(.029)
CP experience (β_4j_)	.093***	(.030)	–.020	(.022)
DIY propensity (β_5j_)	.049	(.035)	.042	(.026)
Gender (β_6j_)	–.036	(.090)	–.102	(.069)
Age (β_7j_)	.007	(.015)	–.009	(.011)
Income (β_8j_)	–.078***	(.026)	.017	(.022)
	DV = Firm-serving CP motives		DV = Customer-serving CP motives	
	γ_i1_	(S.E.)	γ_i2_	(S.E.)
Influence of CP characteristics				
CP design freedom (γ_1j_)	–.002	(.070)	.873***	(.078)
CP intensity (γ_2j_)	.169***	(.069)	–.742***	(.073)
Coproduction savings (γ_3j_)	–.164***	(.069)	.328***	(.072)
Control variables				
Product category involvement (γ_4j_)	–.032	(.027)	–.031	(.034)
CP experience (γ_5j_)	.054**	(.022)	–.010	(.026)
DIY propensity (γ_6j_)	–.013	(.029)	.092***	(.032)
Gender (γ_7j_)	.017	(.074)	–.051	(.082)
Age (γ_8j_)	–.036***	(.012)	.006	(.013)
Income (γ_9j_)	–.040	(.026)	–.004	(.023)

Notes: n = 931; *p < .1; **p < .05; ***p < .01 (two-tailed tests). Estimates show unstandardized coefficients; S.E. = Standard error; CP = Coproduction.

#### Effects on inferred coproduction motives

In support of H5a and H5b, the results show that higher levels of coproduction intensity significantly increase firm-serving motive attributions (H5a: γ_21_ = 0.169, *p* < 0.01) and significantly decrease customer-serving motive attributions (H5b: γ_22_ = –0.742, *p* < 0.01). Furthermore, the results show that higher levels of coproduction design freedom significantly increase customer-serving motive attributions (H6b: γ_12_ = 0.873, *p* < 0.01) but do not significantly reduce firm-serving motive attributions (H6a: γ_11_ = –0.002, *n.s.*), thereby offering support for H6b but not H6a. Similar to the field study’s findings, these results suggest that the degree of design freedom is especially diagnostic for the development of customer- compared to firm-serving motive attributions. Furthermore, we find that higher levels of coproduction savings significantly decrease firm-serving motive attributions (H7a:γ_31_ = –0.164, *p* < 0.01) and significantly increase customer-serving motive attributions (H7b: γ_32_ = 0.328, *p* < 0.01). This finding provides support for H7a and H7b.

#### Ambivalence and dominance of coproduction motive attributions

We employed the same approach as in Study 1 to assess the consequences of a dominance of one motive attribution over the other. Results of this model appear in Web Appendix H and show a significant negative interaction between the extent and the direction of dominance on customer satisfaction (${\beta }_{31}=-.231, p<.01)$ and a negative but a non-significant interaction effect on customers’ willingness to pay more (${\beta }_{32}=-.092,n.s.$). The simple slopes further show that a dominance of firm-serving motive attributions has significantly negative effects (${\omega }_{11}=-.292, p<.01$, ${\omega }_{12}=-.179, p<.01$) whereas the dominance of customer-serving motives has non-significant positive effects on customer satisfaction and willingness to pay more (${\omega }_{21}=.169, n.s.$, ${\omega }_{22}=.004, n.s.$).^6^ Appendix 2, panels 3 and 4, illustrate these effects from weak (1) to strong (6) dominance as compared to equally strong held attributions.

#### Mediation analysis

We conducted a mediation analysis to offer additional insights into the indirect effects of coproduction characteristics on customer outcomes via both customer- and firm-serving motive attributions. Results of the mediation analysis show that the total indirect effects of coproduction intensity (ω_INTCS_ = –0.366, *p* < 0.01; ω_INTWTPM_ = –0.198, *p* < 0.01), design freedom (ω_DFCS_ = 0.404, *p* < 0.01; ω_DFWTPM_ = 0.181, *p* < 0.01), and monetary savings (ω_SAVCS_ = 0.164, *p* < 0.01; ω_SAVWTPM_ = 0.110, *p* < 0.01) are significant and run in the expected direction. Furthermore, all specific indirect effects are in line with the results of the main analysis.

### Discussion

The findings of Study 2 offer further support for our conceptual framework and help to establish the causality of the relationships between coproduction characteristics and inferred firm motives (H5-7). Comparing the results with those of Study 1 reveals two common patterns regarding the relative strength of the effects. First, the effects of firm-serving motive attributions seem to be stronger for willingness to pay more than for customer satisfaction, while the reverse is true for the effects of customer-serving motive attributions on both outcomes. Second, we find that perceived design freedom is a stronger predictor of customer- (vs. firm-) serving motive attributions. While this study provides evidence on the front-end of our model, the following study adds further insights into the generalizability of the full conceptual model.

## Study 3: Cross-company field study

The objective of Study 3 is to provide additional evidence of the robustness of our results at different levels. First, we sampled customers from different firms to capture a broader variety of real market differences in coproduction characteristics with regard to coproduction intensity, design freedom, and monetary savings. This field setting is especially suited to gain more insights into the role of perceived monetary savings in our model. Second, we recruited participants from another European country to assess the generalizability of our findings from a cross-national perspective. We chose the same context (i.e., ready-to-assemble furniture) to facilitate comparison across studies.

### Method

#### Data collection and sample

We administered the study using an online questionnaire. Participants were recruited via the crowdsourcing platform Prolific and received GBP 0.80 for their participation. To make sure that participants had prior coproduction experience, we only invited people that had purchased and assembled a piece of ready-to-assemble furniture within the last two years. The total sample comprises of 360 participants with similar demographic profiles as in our first field study (mean age 38.73, female 73.1%).

#### Procedure

Upon entering the survey, participants indicated from which firm they had last purchased and assembled a piece of ready-to-assemble furniture (37 different firms; e.g., Ikea, Argos, Wayfair, B & M). For each participant, all subsequent questions focused on this specific firm. Participants first evaluated the characteristics of the coproduction concept of the firm and then responded to the items measuring attributions of coproduction motives and customer outcomes. Finally, they responded to the covariate measures and provided demographic information.

#### Measures and psychometric properties

We relied on the same measures to capture our key variables and covariates as in the previous studies (see Appendix 1). We employed a confirmatory factor analysis to assess the psychometric properties of our measures. Results show that our measurement model fits the data well (CFI = 0.93; TLI = 0.93; RMSEA = 0.06; SRMR = 0.08) and that all criteria for reliability and validity are supported (Bagozzi & Yi, 1988).

#### Analytical approach

In line with Study 1 and 2, we relied on a structural equation modeling approach to analyze the data (Kline, 2015). To account for the nested data structure (customers nested in firms), we employed a maximum likelihood estimator that is robust against non-independence and non-normality of observations (Muthén and Muthén 1998–2018).

### Results

#### Effects of inferred coproduction motives on customer outcomes

Our analysis offers further support for H1, as we find significant negative effects of inferred firm-serving motives on customer satisfaction (H1a: β_11_ = –0.126, *p* < 0.05) and willingness to pay more (H1b: β_12_ = –0.302, *p* < 0.01). Analogously, we find significantly positive effects of customer-serving motives on customer satisfaction (H2a: β_21_ = 0.480, *p* < 0.01) and willingness to pay more (H2b: β_22_ = 0.392, *p* < 0.01) that provide additional support for H2 (see Table 4).

**Table 4:** Study 3: Results of multi-firm field study

	DV = Customer satisfaction		DV = Willingness to pay more	
	β_i1_	(S.E.)	β_i2_	(S.E.)
Influence of CP motives				
Firm-serving CP motives (β_1j_)	–.126***	(.037)	–.302***	(.061)
Customer-serving CP motives (β_2j_)	.480***	(.077)	.392***	(.102)
Control variables				
Product category involvement (β_3j_)	.073***	(.021)	.041	(.041)
CP experience (β_4j_)	.104***	(.028)	.100***	(.038)
DIY propensity (β_5j_)	.014	(.017)	.015	(.042)
Customer relationship length (β_6j_)	–.002	(.004)	–.015***	(.005)
CP situation (β_7j_)	.052	(.071)	–.150	(.101)
Gender (β_8j_)	–.125*	(.066)	–.167*	(.092)
Age (β_9j_)	–.006**	(.002)	–.004	(.004)
Income (β_10j_)	.005	(.009)	.017	(.020)
	DV = Firm-serving CP motives		DV = Customer-serving CP motives	
	γ_i1_	(S.E.)	γ_i2_	(S.E.)
Influence of CP characteristics				
CP design freedom (γ_1j_)	–.001	(.034)	.090***	(.033)
CP intensity (γ_2j_)	.349***	(.053)	–.079*	(.042)
Coproduction Savings (γ_3j_)	–.029	(.039)	.437***	(.050)
Control Variables				
Product category involvement (γ_4j_)	–.053**	(.021)	.010	(.027)
CP experience (γ_5j_)	.050***	(.019)	.016	(.033)
DIY propensity (γ_6j_)	.091***	(.021)	–.016	(.027)
Customer relationship length (γ_6j_)	–.010***	(.002)	.001	(.004)
CP situation (γ_7j_)	–.074	(.052)	–.061	(.084)
Gender (γ_7j_)	–.159**	(.074)	–.145**	(.071)
Age (γ_8j_)	–.005	(.004)	–.004	(.003)
Income (γ_9j_)	–.003	(.012)	–.024*	(.013)

Notes: n = 360; *p < .1; **p < .05; ***p < .01 (two-tailed tests). Estimates show unstandardized coefficients; S.E. = Standard error; CP = Coproduction.

#### Effects on inferred coproduction motives

Regarding the effects of coproduction characteristics on inferred firm motives, we find further support for H5 as coproduction intensity has a significantly positive effect on firm-serving motive attributions (H5a: γ_21_ = 0.349, *p* < 0.01) and a marginally significant negative effect on customer-serving motive attributions (H5b: γ_22_ =–0.079, *p* < 0.1). The results also replicate the finding that coproduction design freedom is a stronger predictor of customer-serving motive attributions (H6b: γ_12_ = 0.090, *p* < 0.01) than for firm-serving motive attributions (H6a: γ_11_ = –0.001, *n.s.*), thereby offering support for H6b but not H6a. Finally, we again find that coproduction savings significantly increase customer-serving motive attributions (H7b: γ_32_ = 0.437, *p* < 0.01) while their attenuating effect on firm-serving motive attributions is not significant (H7a: γ_31_ = –0.029, *n.s.*). In sum, these findings closely mirror the pattern of results from Study 1 and 2 and thus add further confidence to our conclusions.^7^

#### Ambivalence and dominance of coproduction motive attributions

We relied on the same approach as in Study 1 and 2, to assess the consequences of one of both motive attributions dominating the other. Results of this model appear in Web Appendix H and show significant negative interaction effects between the extent and the direction of dominance on customer satisfaction and willingness to pay more (${\beta }_{31}=-.099, p<.01$, ${\beta }_{32}=-.138,p<.05$). The simple slopes further show that a dominance of firm-serving motive attributions has significantly negative effects on both outcomes (${\omega }_{11}=-.114, p<.05$, ${\omega }_{12}=-.215, p<.01)$, whereas a dominance of customer-serving motives has a significant positive effect on customer satisfaction (${\omega }_{21}=.084,p<.01)$ and a positive but non-significant effect on willingness to pay more (${\omega }_{22}=.061, n.s.$). Appendix 2, panels 5 and 6, illustrate these effects from weak (1) to strong (6) dominance as compared to equally strong held attributions.

#### Mediation analysis

Results of a mediation analysis show that the expected total and specific indirect effects of coproduction intensity (ω_INTCS_ = –0.052, *p* < 0.01; ω_INTWTPM_ = –0.118, *p* < 0.01), design freedom (ω_DFCS_ = 0.019, *p* < 0.1; ω_DFCSMA_ _CS_ = 0.020, *p* < 0.05; ω_DFWTPM_ = 0.019, *n.s*. ω_DFCSMA_ _WTPM_ = 0.021, *p* < 0.05), and monetary savings (ω_SAVCS_ = 0.108, *p* < 0.01; ω_SAVWTPM_ = 0.119, *p* < 0.1; ω_SAVCSMAWTPM_ = 0.111, *p* < 0.05) run in the expected direction. Moreover, all specific indirect effects are in line with the results of the main analysis.

### Discussion

Study 3 provides further support for the generalizability of our findings pertaining to the effects of inferred firm motives on customer outcomes (H1-2) and coproduction characteristics on inferred firm motives (H5-7). Regarding the back-end of the model, we again find that the effects of firm-serving motive attributions are stronger for willingness to pay more than for customer satisfaction, while the relative importance reverses for the effects of customer-serving motive attributions on both outcomes. Second, we again find that design freedom and monetary savings have stronger effects on customer-serving motive attributions, while coproduction intensity has a stronger effect on firm-serving motive attributions. As these results stem from another European country, the effects seem to be generalizable across Western countries.

## General discussion

The concept of customer participation in the creation of products and services is thriving in the retailing, banking, and traveling industry and is about to transform the health industry in the near future (Mathews et al., 2020; Vargo & Lusch, 2016). However, despite the widespread belief that coproduction creates benefits for both firms and customers (e.g., Atakan et al., 2014; Norton et al., 2012), some studies also document potential negative customer responses to coproduction (e.g., Haumann et al., 2015). In light of these mixed findings and the prevailing cross-sectional perspective in prior research on coproduction (see Dong & Sivakumar, 2017; Shin & Perdue, 2019), the present research provides a novel perspective on coproduction by investigating how consumers process positive and negative perceptions of coproduction and how such diverging perceptions might differ in shaping the development of customer outcomes over time.

Drawing on the multiple inference model of attribution (Reeder et al., 2004) and two qualitative pre-studies, we show that customers attribute ambivalent firm motives for offering coproduction (firm- and customer-serving), which explain how they form and integrate positive and negative coproduction responses. Across three empirical studies, we show that customer-serving motive attributions increase customers’ satisfaction and willingness to pay whereas firm-serving motive attributions diminish both customer outcomes. Notably, these effects are stronger when firm-serving motive attributions dominate customer-serving motive attributions (vs. vice versa). In a longitudinal field study, we further reveal that these effects follow systematically different patterns over time, as the favorable effects of customer-serving motive attributions gradually erode over time while the adverse effects of firm-serving motive attributions are temporarily much more persistent. Beyond the implications for attitudinal customer outcomes, these effects also have substantive downstream consequences for customer spending behavior.

Finally, our research offers first insights into how firm decisions about the degree of customer participation (in the design and realization stage) and the pricing of coproduction offerings affect customers’ attributions of a firm’s underlying coproduction motives. In sum, our research shows that customers’ attributions of firm-serving motives are primarily driven by perceived coproduction intensity in the realization stage, while attributions of customer-serving motives are more strongly affected by perceived freedom to customize the outcome in the design stage and the monetary savings that customers associate with the coproduction offering.

### Theoretical implications

This research makes three major contributions to the academic marketing literature. First, it advances the so far predominantly cross-sectional perspective on coproduction (for a review see Dong & Sivakumar, 2017) by investigating differential short- and long-term consequences of positive and negative coproduction perceptions for customer relationships. Our study is the first to show that customer perceptions of coproduction follow a temporal negativity bias, such that positive effects erode over time while negative effects for customer outcomes are much more persistent over time. This finding challenges the prevailing notion that coproduction experiences generally become less relevant over time when customers become used to these offerings (Wang et al., 2013), by showing that only positive perceptions lose relevance over time, while negative perceptions––once established––are hard to overcome and can damage customer relationships in the long-run.

Second, we advance the sparse knowledge on psychological mechanisms that explain how coproduction shapes customer relationships (Dong & Sivakumar, 2017), by showing that positive and negative perceptions of coproduction can be explained by a dual motive attribution process, based on the multiple inference model of attribution (Reeder et al., 2004). Thus, customers do not perceive a coproduction concept as inevitable but driven by a firm’s strategic motives. While positive attributions of customer-serving motives (e.g., more convenience) lead to reciprocally favorable customer responses in the short run, negative attributions of firm-serving motives (e.g., reduction of service staff) imply an exploitative and unfair approach to the idea of collaborative production (in which “win–win” is more of a euphemism) that can have a long-term damage for customer relationships. These findings also have implications for other business contexts in which inferred firm motives might play a role, such as corporate social responsibility campaigns (e.g., Habel et al., 2016) and sport sponsorships (e.g., Woisetschläger et al., 2017). Thus, by shedding first light on the temporally diverging effects of customer- and firm-serving motive attributions on customer relationship outcomes, we also advance the sparse theoretical knowledge on short- and long-term effects of inferred firm motives in general (Sipilä et al., 2020).

Third, we contribute to an integrated understanding of the related but largely isolated research streams on customer participation in the design and realization stage (Atakan et al., 2014; Buechel & Janiszewski, 2014), by explaining how consumer perceptions of both stages shape their overall relationship with a firm. Here, we add the notion that a firm’s decisions regarding the degree of customer participation in the design (i.e., design freedom) and realization stage (i.e., production intensity), as well as the firm’s pricing of coproduction offerings (i.e., monetary savings), serve as diagnostic cues for customers to infer firm- and customer-serving coproduction motives. With this integrated perspective, our research helps to understand the different conclusions regarding the benefits of coproduction concepts between both literature streams (Dong & Sivakumar, 2017) and advances knowledge on when and why coproduction concepts have positive or negative consequences for customer relationships.

### Managerial implications

The findings of this research offer several important implications for the management and marketing of coproduction concepts. First, whereas coproduction is often praised as an instrument for generating customer loyalty, our studies offer a more nuanced view on its short- and long-term effects on customer satisfaction, willingness to pay, and spending behavior. We show that coproduction can indeed improve customer relationships in the short-run if customers attribute its adoption to a firm’s true intent to improve customer benefits. However, adopting a coproduction concept can also damage customer relationships in the long-run, if customers perceive this decision to be driven by firm-serving motives. To quantify the monetary consequences of these effects we also ran an additional analysis, which shows that customers who attribute very high (vs. very low) firm-serving motives on average generate around 12.66% lower sales volumes over the period of our study. Although this figure may vary depending on the specific context and coproduction concept, it offers a first starting point for firms to assess the long-term costs and benefits of adopting a coproduction concept (Shin & Perdue, 2019).

For managers, these findings highlight the relevance of striking a balance between firm- and customer-serving interests when adopting a coproduction concept. This is especially relevant as consulting reports show that business executives substantially overestimate the benefits of coproduction for their customers (Detecon Consulting, 2014). Our results on the dominance of firm-serving (over customer-serving) motive attributions highlight the potentially detrimental consequences of such misperceptions. The pitfalls of over-focusing on firm-serving benefits are also observed in prominent business cases in which large retail chains such as Walmart, Home Depot, and Albertsons had to retract self-checkout systems in light of decreasing customer satisfaction and spending rates (Bloomberg, 2018; Gizmondo, 2019). Therefore, to avoid implementing a concept that customers perceive to be driven more by firm- rather than customer-serving interests, it would thus be more advisable for firms to test new concepts on a small scale and closely listen to customers’ feedback rather than releasing a concept in a large national rollout after which any changes are more expensive. Moreover, firms should constantly review and improve existing concepts to demonstrate their care for customers.

Our findings also have implications for marketing communications. To advertise their coproduction concepts, firms often rely on claims that suggest purely customer-serving motives for adopting a coproduction concept. These claims are especially prevalent in the retail and travel industry, where many providers advertise their self-service concepts as great opportunities for customers to save time (American Airlines, 2020) or means to shop more independently and conveniently (Walmart, 2020), while keeping firm-serving motives like cutting down labor costs below the line (Bloomberg, 2018). Although this strategy might seem intuitive for marketers, concealing existing firm interests might make customers even more suspicious about a firm’s self-serving motives (e.g., Campbell, 1995; Forehand & Grier, 2003). By raising awareness for the temporal stability with which firm-serving motive attributions harm customer relationship outcomes over time, our research extends managerial insights into the potentially detrimental long-term consequences of such a communication strategy.

Beyond implications for marketing communication, our studies offer clear guidance for managers who want to understand how their decisions related to the selection of specific features of a coproduction concept influence the success of the customer relationship. Here, we show that more intense customer participation in the realization stage reduces customers’ satisfaction and willingness to pay for the firm’s products, which can have sustained negative consequences for customer relationships over time (by increasing firm-serving motive attributions). Managers must therefore carefully weigh the internal cost savings that result from outsourcing efforts and time-consuming production tasks to customers against the negative impact of this decision on the long-term development of successful customer relationships.

One remedy to reduce the negative short- and long-term effects of coproduction intensity is to invest in options that allow customers to have more freedom in designing the coproduction outcome and thereby increase attributions of customer-serving coproduction motives. This could be accomplished by increasing the variety of input options (e.g., offering more design options), offering customization toolkits (e.g., digital product planer), or providing customers with more inspiration for alternative outcome options (e.g., different outcome versions that can be achieved with the same input product). A second remedy is a more direct compensation of customers’ coproduction efforts in terms of monetary savings that customers can realize by participating in coproduction (Xia & Suri, 2014), which reduces attributions of firm-serving motives and heighten attributions of customer-serving motives. Such monetary savings could be signaled using internal (e.g., by displaying the costs of full-service options) or external price comparisons (e.g., by facilitating comparisons with firms that do not offer coproduction options). In sum, our study provides a first integrative view on these three factors that help managers to understand when and why coproduction leads to positive or negative implications for customer relationships.

### Limitations and future research directions

Although we provide robust evidence for the proposed effects across three studies, this work is not without limitations that point to avenues for future research. We conducted our studies in the context of ready-to-assemble furniture, as this is one of the most prototypical coproduction settings (e.g., Bendapudi & Leone, 2003). While this approach allows us to compare results across studies, a limitation is that we are not able to draw direct comparisons to other contexts involving customer participation, such as self-checkout or check-in systems. As market norms regarding the level of intensity, design freedom, and monetary savings may vary across contexts, an open question is whether the proposed inference model changes when certain market standards cross thresholds of acceptance across a larger proportion of the population. Relatedly, it would be interesting to explore how the lack of a common market standard for coproduction might affect the proposed inference model. Future work could use large-scale experimentations (e.g., Bleier et al., 2019) or conjoint designs to test these ideas and provide more fine-grained management implications about the ‘optimal configuration’ of coproduction concepts across specific contexts.

We focus on customer satisfaction and willingness to pay as two of the most frequently studied outcomes in prior coproduction research (Dong & Sivakumar, 2017). However, these outcomes do not allow us to make predictions about when customers actually decide to leave or switch to another firm because of a coproduction concept. Examining when inferred coproduction motives can trigger such a “tipping point” in the customer relationship is thus an interesting open question for further research. Moreover, future research could also offer more insights into how adapting a coproduction concept affects other relevant customer outcomes, such as perceived relational value (Singh, Sirdeshmukh, and Sabol 2002), customer value (Zeithaml et al., 2020), or customer engagement (Harmeling et al., 2017).

As we conducted our studies in two Western countries, another fruitful avenue for future research is to explore whether the identified temporal dynamics would also hold in other cultural contexts or market environments with different expectations regarding the level of customer service. As there is yet little work on how cultural or market-related factors influence the effects of coproduction on customer relationships (Nilsson, 2007), these ideas offer interesting starting points for future research.

### Electronic supplementary material

Below is the link to the electronic supplementary material.

Supplementary file1 (PDF 813 kb)

## Appendix 1

**Table:** Measurement scales

*Firm-serving motive attributions* Adapted from Ellen et al. (2006), Nijssen et al. (2016), and Vlachos et al. (2009) What do you think are the motives of [firm name] underlying their self-assembly concept? [Firm name] pursues the self-assembly concept… 1. to generate more profits 2. to take advantage of it 3. because it offers benefits for [firm name] 4. to let customers do the work 5. to make more profits instead of serving customers *Customer-serving motive attributions* Adapted from Ellen et al. (2006), Nijssen et al. (2016), and Vlachos et al. (2009) What do you think are the motives of [firm name] underlying their self-assembly concept? [Firm name] pursues the self-assembly concept… 1. because it meets the needs of their customers 2. as this concept offers many advantages for customers 3. because it offers benefits for their customers 4. to make shopping for furniture more fun for their customers 5. to allow customers to save money when shopping for furniture *Customer satisfaction* Homburg et al. (2006) 1. Overall, I am satisfied with [firm name] 2. [Firm name] is the ideal furniture store 3. Taking into account all aspects that are related to buying furniture, I am very satisfied with [firm name] *Willingness to pay more* Adapted from Zeithaml et al. (1996) and Srinivasan et al. (2002) 1. I would be willing to pay higher prices at [firm name] than at a competitor 2. I would continue doing business with [firm name] even if other competitors would offer lower prices 3. I would pay a higher price at [firm name] relative to the competition for the same benefit *Design freedom* Adapted from Spreitzer (1995) How do you evaluate the opportunities to customize the design of ready-to-assemble furniture at [firm name]? 1. I have a significant influence on the specification of products at [firm name] 2. [Firm name] offers its customers a great deal of control over the configuration of products 3. My impact on the design of products at [firm name] is large *Coproduction intensity* Adapted from Franke and Schreier (2010), Franke et al. (2010), and Troye and Supphellen (2012) How do you evaluate the process of assembling furniture from [firm name]? Assembling products of [firm name] is … 1. effortful 2. exhausting 3. demanding 4. time-consuming 5. costly (in terms of time and effort) *Monetary savings* Given the self-assembly concept for furniture at [firm name], how do you evaluate their prices? 1. Firm name] offers products with a favorable cost–benefit ratio 2. Purchasing products from [firm name] offers a good value for money 3. The prices at [firm name] are more attractive than at other furniture retailers *DIY propensity* Adapted from Bateson (1985) and Williams (2008) 1. I am a DIYer 2. I like to do DIY projects at home 3. Engaging in DIY projects is fun *Product category involvement* Adapted from Zaichkowsky (1985) 1. I am generally interested in furniture 2. Buying furniture is important for me *Prior Coproduction experience* Adapted from Dellaert and Stremersch (2005) • I have a lot of experience in assembling furniture	

All items were measured using seven-point Likert-scales anchored at 1 = “strongly disagree” to 7 = “strongly agree”. DIY = do-it-yourself

## Footnotes

1 Although we assume the mechanisms we study to be generalizable across different types of coproduction, our empirical focus is mainly on coproduction concepts involving tangible outcomes.

2 Note that this study does not include a measure of perceived monetary savings and thus only allows us to test H1-6. We test H7 in Study 2 and 3 and perform an additional analysis to show that the omission of monetary savings in Study 1 is unlikely to affect its results and implications.

3 We employed a scheme for coding time in that the intercepts reflect the level of customer satisfaction and willingness to pay more at t_0_ and the slope parameters reflect the changes in these marketing outcomes across the timeframe of the data collection period.

4 Due to the marginally significant effect of customer-serving motive attributions on the latent slope factor of the growth trajectory of willingness to pay more, the indirect effects via this path are also marginally significant (*p* < .10, two-tailed; cf. Table WI.1 in Web Appendix I).

5 In light of the direct effects (see Web Appendix L), we thus find support for full mediation for the effects of firm-serving coproduction motive attribution, CP design freedom, and intensity on customer spending and partial mediation for customer-serving coproduction motive attribution.

6 Simple slopes for willingness to pay more are only included for illustrative purposes as the interaction effect is not significant.

7 We additionally tested if and how our results are affected by prior experience in design freedom (i.e., a dummy that captured whether customers have any prior experience in customizing ready-to-assemble furniture at the focal firm). The results show that prior experience in design freedom positively affects customers’ perceived design freedom (γ_DFE__→__DF_ = 1.509, *p* < .05), but does not have a moderating effect on the relationships between the characteristics of the coproduction concept and both motive attributions (except a marginally significant interaction between design freedom experience and coproduction intensity on firm-serving motive attributions; γ_DFExINT__→__FSMA_ = .-113, *p* < .10). In sum, these results indicate that prior experience in design freedom does not structurally affect the proposed relationships.
