# Supplementary material for: How attributions of coproduction motives shape customer relationships over time
Source: J Acad Mark Sci. 2023 Jan 14:1–29. Online ahead of print. doi: 10.1007/s11747-022-00910-6 (PMC9839446; doi:10.1007/s11747-022-00910-6)
Supplement: Supplementary file 1 — Supplementary file1 (PDF 813 kb) [file 11747_2022_910_MOESM1_ESM.pdf]

Web Appendix

How Attributions of Coproduction Motives Shape Customer Relationships over Time

Web Appendix A

Qualitative Pre-Studies on the Prevalence and Content of inferred Coproduction Motives

We conducted two qualitative pre-studies to provide first systematic evidence on the prevalence of inferred firm motives across different coproduction contexts. In both pre-studies, we exposed participants to a number of typical examples of coproduction concepts and asked them to reflect on their perceptions about the reasons that drive a firm's decision to rely on such a concept spontaneously. The questions were unaided and in an open-ended question format requiring participants to enter their perceptions in their own words. In Pre-Study A, participants answered this question more generally, while participants in Pre-Study B were specifically asked about their perceptions in three prototypical coproduction contexts (i.e., furniture self-assembly concept, self-checkouts in grocery stores, self-check in kiosks at airports and hotels). Independent coders who were blind to our hypotheses categorized the responses into reasons that either reflect firm- and/or customer-serving motives.

Qualitative Pre-Study A

Sample and data collection. The study was distributed in a paper-and-pencil format among business administration students at a large public university. Sixty-three students participated in exchange for course credits (mean age 23.3, female 38%).

Procedure. Participants first read that the study is about situations in which customers are actively involved in the production of goods and services. To establish a common understanding of the concept, participants were also exposed to illustrative examples of prototypical coproduction contexts (i.e., furniture self-assembly concept, self-checkouts in grocery stores, self-check in kiosks at airports and hotels). We then asked participants to indicate their perceptions about the main reasons that drive firms' decision to rely on a coproduction concept. Participants wrote their answers in an open field, provided demographics (age, gender), and were debriefed.

Analysis. We received 167 different answers for the open question, resulting in an average of 2.65 motives per participant. Two research assistants who were blind to the aim of the study and its hypotheses independently rated each answer with regard to its association with a benefit for the firm and/or the customers of a firm. The interrater-agreement was high (r= .92) and unclear cases were resolved in discussion. Raters further identified subcategories of these benefits to offer further insights into the specific firm and customer benefits.

Results. The results clearly indicate that consumers view a firm's motivation for engaging customers in coproduction as driven by both benefits for the firm and benefits for customers. The reasons associated with benefits for the firm mostly refer to cost savings and efficiency gains and thus closely match our definition of firm-serving motives. The reasons associated with benefits for customers capture time and money savings, a better user experience, convenience and autonomy, and a stronger bond with the resulting product. These motives clearly resemble a firm's customer-serving motives. Notably, the results show that participants mentioned significantly more firm- (55%) than customer-serving motives (45%) ($Chi^2 = 4.618$, $p < .05$).

Discussion. In sum, Pre-Study A supports our core assumption that consumers' believes that a firm's decision to rely on a coproduction concept is driven by both firm- and customer-serving motives. Notably, the results further suggest that inferred firm motives are more complex than suggested in prior studies on coproduction (Dong and Sivakumar 2018), as consumers view these two motives as conceptually distinct and operating in parallel. Overall, the study also supports the importance of the role of firm benefits from a consumer's perspective, as consumers mentioned firm benefits (55%) more frequently than customer benefits (45%).

Qualitative Pre-Study B

Sample and data collection. In Pre-Study B, we aimed to gain more insights into consumers' attribution of firm motives across different types of coproduction concepts. We administered the study using an online survey. The study was part of a subject pool at a large public university. Two hundred eleven undergraduate students participated in exchange for course credits. We excluded seven participants because they failed to complete the study, resulting in a total sample of 204 participants (mean age 21.2, female 52%).

Procedure. Participants first read that the study is about situations in which customers are actively involved in the production of goods and services. Each participant was set out to evaluate two different coproduction settings. We randomly assigned each participant to two of the three settings and randomized the sequence to minimize potential ordering effects. We briefly described each coproduction situation to make sure that participants were familiar with the context. We avoided making any connections to actual brands or service providers. We then asked participants to share their perceptions about the three main reasons as to why [furniture retailers; grocery stores; airlines] increasingly rely on a coproduction concept.

Analysis. We received 354 [345; 328] answers for the open question in the ready-to-assemble furniture [self-checkout; self-check in] context from 136 [135; 135] respondents, resulting in an average of 2.6 [2.6; 2.4] motive attributions per participants. Two teaching assistants blind to the purpose of the study independently coded the motives for each context regarding whether they benefit the firm and/or its customers and further identified categories of common responses. The interrater-reliability was high ($r = .95$), thus suggesting that the resulting categories were reliable and clear.

Results. The results in all contexts clearly support the notion that consumers view a firm's motivation for engaging customers in coproduction as driven by both benefits for the firm and benefits for customers. Figure 2 provides illustrative examples of the motives mentioned in each context. Clearly, the most frequently mentioned firm motive across all three contexts is cost savings (88%, 53%, and 56% respectively), a finding that offers strong

support for the notion that firm benefits are indeed salient for consumers in coproduction situations. Regarding customer-serving firm motives, customers also mention lower prices for customers in furniture self-assembly (39%) and saving time in the context of self-checkout (68%) or self-check in kiosks (75%).

In line with Pre-Study A, we again find that customers name significantly more firm- (59%) as compared to customer-serving motives (41%) ($X^2 = 61.64$, $p < .01$). However, the data of Pre-Study B enable us to gain a more differentiated view on the differences across the three considered coproduction concepts. Regarding the frequency of mentions, we find no difference between firm- (FS) and customer-serving (CS) motive attributions in the ready-to-assemble furniture context (FS = 51%; CS = 59%; $X^2 = .18$, *n.s.*). However, in the self-checkout (FS = 62%; CS = 38%; $X^2 = 20.40$, $p < .01$) and self-check in contexts (FS = 63%; CS = 37%; $X^2 = 22.55$, $p < .01$), participants mentioned significantly more firm- than customer-serving motives (see Figure 2).

Discussion. Pre-Study B demonstrates that consumers believe that a firm's decision to rely on coproduction is driven by both firm- and customer-serving motives across different types of coproduction concepts. Firm-serving motive attributions are mainly associated with a firm's striving for cost savings, whereas customer-serving motive attributions are mostly associated with reduced prices (in the self-assembly context) and time savings (in the SST contexts), but also include convenience, autonomy, and other benefits that these concepts imply for customers. Notably, while the frequency of firm- and customer-serving motive attributions was balanced in the furniture self-assembly context, consumers seem to attribute significantly more firm-serving motives in both SST contexts. This finding suggests that the context of furniture self-assembly might offer a rather conservative setting for investigating the consequences of such motive attributions.

Web Appendix B
Study 1 – Tests for Nonresponse

We ran several tests to assure that nonresponse is not an issue in our data and that our dataset is representative for the firm's customer base. First, we tested whether early and late respondents differ with regard to their responses to key study constructs and demographic variables across all waves. The results show no significant differences on any of the variables. Second, we tested whether respondents and 1) non-participants (i.e., customers who received an invitation to participate but declined) and 2) non-surveyed customers (i.e., customers who did not receive an invitation to participate) differ in terms of key demographics (i.e., gender) and customer spending behavior. Third, we asked our contact at the focal company for a qualitative assessment of whether the respondents in our sample are representative of the firm's customer profile and reflective of the typical customer profile in the specific industry.

Respondents vs. non-participants. We performed a chi-square test to assess potential gender differences and an ANOVA to assess potential differences in customer spending behavior between respondents and non-participants. The results of a chi-square test show that there are no statistically significant gender differences between both groups (X^2 = .615, p = .433). Moreover, the results of the ANOVA show that respondents and non-participants did not significantly differ in terms of their prior spending behavior (F = 1.465, p = .226).

Respondents vs. non-surveyed customers. As we do not have access to the company's database (all customers in our dataset were invited to participate in the study), we compared respondents with customers who should have, but did not, receive an invitation to the study due to an email delivery failure (e.g., connection timed out, mailbox unavailable, unknown recipient, etc.). A chi-square test shows that there are no statistically significant gender differences between both groups (X^2 = 2.631, p = .105). The results of the ANOVA show that both groups do not significantly differ in terms of prior spending behavior (F = .360, p = .548).

Sample representative for firm's customer profile. Finally, we also asked our contact at the company (head of marketing research responsible for the respective country) to assess whether respondents are representative of the firm's customer profile. He confirmed that this is indeed the case. Moreover, he indicated that the surveyed population is also representative of the typical customer profile in the industry. The highly similar demographic profiles of respondents in Study 1 (77.7% female, mean age 37.85) and Study 3 (73.1% female; mean age = 37.85) provide additional insights into the external validity of this assessment from a cross-cultural perspective.

In sum, the additional tests clearly indicate that there are no significant differences between respondents and 1) non-participants and 2) non-surveyed customers with regard to key demographics (i.e., gender) and customer spending behavior. The results of these tests indicate that nonresponse is not an issue in our data and that respondents are representative of the firm's customer profile.

Web Appendix C

Study 1 – Correlations and Psychometric Properties of Measures

Variable	1	2	3	4	5	6	7	8	9	10	11	12	13	14	15	16	17	18	19	20	21	22	23
1. Firm-serving CP motives	(.88)																						
2. Customer-serving CP motives	-.010	(.84)																					
3. Customer satisfaction (t_0)	-.121**	.614**	(.91)																				
4. Customer satisfaction (t_1)	-.153**	.571**	.787**	(.92)																			
5. Customer satisfaction (t_2)	-.127**	.498**	.765**	.805**	(.90)																		
6. Customer satisfaction (t_3)	-.136**	.470**	.693**	.773**	.795**	(.93)																	
7. Customer satisfaction (t_4)	-.142**	.487**	.720**	.756**	.770**	.774**	(.93)																
8. Customer satisfaction (t_5)	-.142**	.491**	.691**	.712**	.767**	.731**	.783**	(.94)															
9. Willingness to pay more (t_0)	-.172**	.309**	.383**	.379**	.363**	.343**	.363**	.348**	(.94)														
10. Willingness to pay more (t_1)	-.159**	.311**	.375**	.408**	.372**	.352**	.378**	.364**	.682**	(.95)													
11. Willingness to pay more (t_2)	-.166**	.294**	.375**	.374**	.406**	.379**	.389**	.331**	.666**	.681**	(.94)												
12. Willingness to pay more (t_3)	-.178**	.297**	.356**	.360**	.388**	.392**	.400**	.362**	.645**	.688**	.726**	(.94)											
13. Willingness to pay more (t_4)	-.145**	.311**	.357**	.346**	.368**	.346**	.420**	.375**	.634**	.639**	.713**	.750**	(.95)										
14. Willingness to pay more (t_5)	-.153**	.269**	.329**	.334**	.348**	.349**	.387**	.365**	.644**	.661**	.686**	.727**	.721**	(.96)									
15. CP design freedom	-.095**	.412**	.426**	.436**	.393**	.372**	.389**	.381**	.249**	.259**	.238**	.278**	.265**	.241**	(.80)								
16. CP intensity	.154**	-.298**	-.322**	-.317**	-.279**	-.289**	-.282**	-.256**	-.188**	-.185**	-.170**	-.185**	-.179**	-.165**	-.190**	(.93)							
17. Product category involvement	-.032*	.239**	.293**	.294**	.312**	.245**	.278**	.299**	.156**	.173**	.163**	.147**	.146**	.148**	.315**	-.100**	(.86)						
18. DIY propensity	-.008	.270**	.205**	.219**	.167**	.185**	.193**	.159**	.175**	.180**	.150**	.184**	.202**	.187**	.315**	-.307**	.201**	(.92)					
19. CP experience	.097**	.221**	.187**	.156**	.149**	.141**	.149**	.152**	.111**	.113**	.097**	.116**	.150**	.103**	.149**	-.246**	.192**	.496**	–				
20. Customer relationship length	.032**	-.018†	-.018†	-.022†	-.007	-.022	-.035*	-.013	-.022*	-.032*	-.019	-.001	.015	-.021	-.046**	-.009	-.013	-.001	.051**	–			
21. Gender	.097**	-.117**	-.135**	-.134**	-.109**	-.109**	-.133**	-.118**	-.025*	-.033**	-.029*	-.025†	-.043**	-.012	-.092**	.016	-.223**	.107**	.113**	-.034**	–		
22. Age	.003	.007	-.020†	-.016	-.020	-.029*	-.029*	-.039*	-.058**	-.090**	-.091**	-.092**	-.062**	-.066**	-.092**	.070**	-.153**	-.084**	-.097**	.114**	.181**	–	
23. Income	.074**	-.024*	-.076**	-.067**	-.058**	-.044**	-.060**	-.060**	-.043**	-.050**	-.051**	-.044**	-.066**	-.070**	-.075**	.040**	-.027*	-.053**	.019*	.115**	.133**	.155**	–
**Mean**	5.31	5.45	5.72	5.62	5.63	5.55	5.58	5.70	3.21	3.27	3.24	3.38	3.61	3.30	5.37	3.10	5.79	5.19	5.38	6.22	.22	37.85	5.41
**Standard deviation**	1.37	1.23	1.06	1.10	1.05	1.09	1.15	1.06	1.58	1.61	1.56	1.58	1.63	1.65	1.14	1.33	1.11	1.62	1.52	2.74	.42	10.71	1.85
**Composite reliability**	.88	.85	.92	.93	.91	.93	.94	.94	.94	.95	.94	.95	.95	.95	.81	.92	.87	.92	–	–	–	–	
**Average variance extracted**	.71	.65	.79	.81	.77	.83	.83	.83	.85	.87	.84	.85	.87	.87	.59	.69	.76	.80	–	–	–	–	

Notes: * $p < .05$, ** $p < .01$, † $p < .10$; Cronbach's (1951) internal consistency reliability coefficients appear in parentheses on the diagonal; CP = coproduction; gender: 0 = female, 1 = male; time elapsed since CP = Number of days elapsed since the last CP; income ranging from (1 = <500€, to 8 = >3500€). Except customer relationship length, gender, age, and income all items were measured on seven-point Likert scales. Additional time-varying control variables not presented for ease of interpretation.

Web Appendix D

Study 1 – Tests for Common Method Bias

Multiple conceptual reasons and results of additional statistical tests make it unlikely that the results and conclusions of the study are driven by common method variance. First, longitudinal survey data are generally less susceptible to common method bias because of the time lag between measurement waves (Podsakoff et al. 2003). Second, we assess the validity of our measures by providing additional insights into their relationships with objective firm data on customer spending (see additional analyses section). Third, we find support for theoretically plausible interaction effects; a finding that further indicates that common method variance is not an issue in our data (Siemsen, Roth, and Oliveira 2010).

Although these aspects reduce the susceptibility of our findings to common method bias, we additionally conducted three statistical tests to rule out potential issues of common method variance empirically. First, we conducted a confirmatory factor analytical approach to Harman's single factor test (Griffith and Lusch 2007; Olson, Slater, and Hult 2005). This test assesses whether a single latent factor can account for all latent variables (Podsakoff et al. 2003). A worse fit of the one-factor model as compared to the original measurement model would indicate that common method bias does not pose a serious threat (Olson, Slater, and Hult 2005; Sanchez, Korbin, and Viscarra 1995). Results of this test show that the fit of one-factor model is poor (e.g., CFI = .391; TLI = .245) and significantly worse than that of the original measurement model. This finding offers first indications that common method bias is not a serious problem in the analyses.

Second, in acknowledgement of certain limitations of Harman's single factor test, we employed the marker-variable approach developed by Lindell and Whitney (2001) to additionally assure that common method variance does not bias the results. This approach suggests that the lowest positive correlation between a theoretically unrelated marker variable and an endogenous construct of the model is indicative of common method variance (Lindell

and Whitney 2001). If partialling out this correlation from other correlations does not affect their significance, biased results due to common method variance are unlikely (e.g., Grayson 2007; Sheng et al. 2011). In line with previous research, customers' age was identified as a marker variable (Griffith and Lusch 2007) as it is theoretically unrelated to the core variables of the model. Using the lowest positive correlation between the marker variable and one of the core variables (i.e., firm-serving motives, r = .003, *n.s.*) to adjust the other correlations does not change the significance of any correlation between the core variables of the model. This result offers further support that common method bias is not a major issue in the data.

Third, common method bias was assessed using an unmeasured latent method factor (Podsakoff et al. 2003). Comparing the results of the original measurement model to a confirmatory factor analysis in which each indicator variable additionally loads on the latent method factor shows that the average variance explained in the indicator variables increases by less than 1.5%. Furthermore, including the unmeasured latent factor does not change the significance of any of the correlations between the study's focal constructs, which further indicates that common method bias is not a problem. Overall, these three additional tests offer additional evidence that common method bias is not a threat to the results and conclusions of this study.

Furthermore, to rule out that the effects of customer-serving and firm-serving coproduction motive attributions on the intercept parameters of growth trajectories are unduly influenced by common method variance, we conducted an additional analysis in which we did not include the measures of customer satisfaction and willingness to pay more of the first wave (i.e., at t = 0) in the estimation of the growth trajectories. Thus, in this model, the effects of both types of coproduction motive attributions (measured at t = 0) are tested on the latent intercept factor of the growth trajectory (at t = 1). Results of this model are in line with those from the original model, thereby offering further support for the robustness of our findings and providing further indications that our findings are not driven by common method variance.

Web Appendix E

Study 1 – Additional Details of Model Specification of Latent Growth Analysis

Unconditional latent growth models. Before estimating the main model of Study 1, we estimated unconditional models for customer satisfaction and willingness to pay more, to assess the variances of the intercept and slope parameters of the growth trajectories which may then be explained by adding the predictors to the model. Indeed, results show significant variance in the intercept and slope parameter of the customer satisfaction trajectory ($\psi_{Intercept,CS} = .666, p < .01$, $\psi_{Slope,CS} = .189, p < .01$) and significant variance in the intercept and slope parameter of the willingness to pay more trajectory ($\psi_{Intercept,WTPM} = 1.481, p < .01$, $\psi_{Slope,WTPM} = .418, p < .01$). These findings additionally empirically motivate the inclusion firm- and customer-serving coproduction motive attributions as predictors to explain the variances in the intercept and slope parameters of the growth trajectories.

Residual structures. Before estimating our main model, we additionally tested for differences in model fit between hetero- and homoscedastic residual structures for linear and quadratic growth curve models. Results in Table WE.1 show that the unconditional models for customer satisfaction and willingness to pay more fit well irrespective of the assumed residual structure. Differences in model fit indices between homo- and heteroscedastic residual structures are negligible for models for customer satisfaction (CS) and willingness to pay more (WTPM). Nested model comparisons reveal no significant differences between heteroscedastic and homoscedastic residual structures (CS: $\Delta\chi^2 = 0.151, n.s.$; WTPM: $\Delta\chi^2 = 1.865, n.s.$).

Growth patterns. To motivate the estimation of a latent growth curve model, we first tested for significant growth (vs. no growth) in customer satisfaction and willingness to pay more over time. Evaluating the unconditional linear growth curve models show significant mean slope parameter both for the customer satisfaction ($\mu_\alpha = -.049, p < .01$) and for the willingness to pay more model ($\mu_\alpha = .176, p < .01$). These findings reject the null hypotheses of no growth (Bentein et al. 2005).

We additionally compared the linear growth model to a quadratic growth model. Comparing the fit indices between both growth models show no substantial differences between linear growth and quadratic growth (see Table WE.2). The only significant differences in model fit can be found in the Chi-Square difference tests. However, these tests are known to be sensitive to distributional assumptions and large sample sizes (Bollen and Curran 2006, Preacher et al. 2008). This sensitivity could be especially problematic given the large data set (n = 12,662) in Study 1. For reasons of parsimony and ease of interpretation we, therefore, opted for a linear growth model rather than a quadratic growth model.

Nevertheless, we additionally tested our model assuming quadratic growth. First, we estimated a model in which we include a quadratic slope parameter for the customer satisfaction and willingness to pay more trajectory, but do not predict these growth parameters. Results in Table WE.3 show that assuming quadratic growth does not substantially affect our results and hypotheses tests. Second, we estimated a model in which we predicted both slope parameters, which however lead to over-specification of the willingness to pay trajectory. We, therefore, estimated a third model in which we included both quadratic growth parameters, but only predicted the quadratic growth parameter for customer satisfaction (and not for willingness to pay more). Results appear in Table WF.4. Again, results remain stable. In addition, we find a small quadratic effect of customer-serving motive attribution on the quadratic slope factor of the customer satisfaction trajectory, indicating that the positive effect of customer-serving motive attributions decreases at a slightly decreasing rate.

Further details on the specification of the main model (Table 1). In estimating our main model, we followed general recommendations in latent growth modeling (Bollen and Curran 2006) and opted for a dual-process second-latent growth model in which we estimate one growth process for customer satisfaction and one for customers' willingness to pay more.

Each growth process is characterized by one latent intercept (α_i) and one latent slope factor (β_i). The latent intercept and slope factor of the customer satisfaction trajectory are informed by the latent measurements of customer satisfaction across the six waves, each

measured with three items. The latent intercept and slope factors of the willingness to pay more trajectory are informed by the latent measurements of willingness to pay more across the six waves, each measured with three items. Due to repeated latent measures design, disturbances of like items were allowed to covary over time (Ployhart and Vandenberg 2010; Sayer and Cumsille 2001, Williams, Edwards, and Vandenberg 2003). Reflecting the measurement invariance tests (Ployhart and Vandenberg 2010; Chan 1998), we specified the model to reflect full configural, metric, and scalar invariance for both customer satisfaction and willingness to pay more latent measures.

We employed a time-coding scheme so that the intercepts reflect the level of customer satisfaction and willingness to pay more at t = 0 and the slope parameters reflect the changes in these marketing outcomes across the timeframe of the data collection period (32 weeks). Weights were chosen to reflect the specific time of the measurement of each wave (Biesanz et al. 2004).

Furthermore, we allowed the latent intercept and slope parameters to covary. As core predictors, we included customers' attributions of customer-serving and firm-serving coproduction motive attributions as well as all time-invariant control variables at t = 0. Additionally, we include 5 time-varying controls in each wave (i.e., whether customers moved between waves, reception of positive WoM and/or news coverage, reception of negative WoM and/or news coverage, positive experiences with the company, negative experiences with the company). All time-invariant controls were allowed to covary with each other and the time-varying controls (Kline 2005). All time-varying controls were additionally allowed to covary over time. Moreover, following established procedures in latent growth modeling (e.g., Bollen and Curran 2006), we constrained the influence of each time-varying control variable over time. Tests of hypotheses remain stable irrespective of including these constraints.

Table WE.1
Study 1: Comparison of Model Fit for Different Residual Structures

Growth Trajectory	Residual Structure	RMSEA	RMSEA 90% CI	CFI	TLI	SRMR
**Customer Satisfaction**	Heteroscedastic	.04	[.042; .044]	.94	.93	.06
	Homoscedastic	.04	[.040; .042]	.94	.94	.06
**Willingness to Pay More**	Heteroscedastic	.03	[.027; .030]	.97	.97	.03
	Homoscedastic	.03	[.026; .028]	.97	.97	.03

Table WE.2
Study 1: Comparison of Model Fit for Different Functional Forms of Growth

Growth Trajectory	Functional Form	RMSEA	RMSEA 90% CI	CFI	TLI	SRMR
**Customer Satisfaction**	Linear	.04	[.042; .044]	.94	.93	.06
	Quadratic	.04	[.042; .045]	.94	.93	.04
**Willingness to Pay More**	Linear	.03	[.027; .030]	.97	.97	.03
	Quadratic	.03	[.027; .030]	.97	.97	.03

Table WE.3
Study 1: Core Results of Dual-Process Latent Growth Analyses with Unconditional Quadratic Growth Parameters

	Main Effects Model			
	DV = Customer Satisfaction		DV = Willingness to Pay More	
	Intercept β_{i1} (S.E.)	Linear Slope β_{i2} (S.E.)	Intercept β_{i3} (S.E.)	Linear Slope β_{i4} (S.E.)
**Influence of inferred CP Motives**				
Firm-serving CP motives (β_{1j})	–.042*** (.006)	–.007 (.011)	–.158*** (.018)	.022 (.021)
Customer-serving CP motives (β_{2j})	.388*** (.013)	–.134*** (.021)	.301*** (.022)	–.061* (.032)
**Difference between CP Motive Effects**				
Firm-serving CP motives –				
Customer-serving CP motives ($\lvert\beta_{1j}\rvert - \lvert\beta_{2j}\rvert$)		–.127*** (.024)		–.039 (.036)

	DV = Firm-Serving CP Motives	DV = Customer-Serving CP Motives
	γ_{i1} (S.E.)	γ_{i2} (S.E.)
**Influence of CP Characteristics**		
CP design freedom (γ_{1j})	–.089*** (.021)	.309*** (.018)
CP intensity (γ_{2j})	.208*** (.014)	–.180*** (.012)

Notes: *p < .1; **p < .05; ***p < .01 (two-tailed tests). Please note that the estimated model include linear and quadratic growth parameters for both outcome variables. Estimates show unstandardized coefficients; S.E. = Standard error; CP = Coproduction. Included control variables: Product category involvement; CP experience; CP situation; customer relationship length; DIY propensity; gender; age; income; reception of positive word of mouth and/or news coverage about focal company ($t_{0\text{-}5}$), reception of negative word of mouth and/or news coverage about focal company ($t_{0\text{-}5}$), positive experience with focal firm ($t_{0\text{-}5}$), negative experience with focal firm ($t_{0\text{-}5}$), indicator of whether a customer moved within the last 6 weeks ($t_{0\text{-}5}$). Standard errors of differences in effect sizes are based on multivariate delta method (e.g., Bishop, Fienberg, and Holland 1975).

Table WE.4
Study 1: Core Results of Dual-Process Latent Growth Analyses Predicting Quadratic Growth for the Customer Satisfaction Trajectory

	Main Effects Model				
	DV = Customer Satisfaction			DV = Willingness to Pay More	
	Intercept β_{i1} (S.E.)	Linear Slope β_{i2} (S.E.)	Quadratic Slope β_{i3} (S.E.)	Intercept β_{i3} (S.E.)	Linear Slope β_{i4} (S.E.)
**Influence of inferred CP Motives**					
Firm-serving CP motives (β_{1j})	–.090*** (.020)	–.018 (.030)	.002 (.033)	–.158*** (.013)	.019 (.021)
Customer-serving CP motives (β_{2j})	.394*** (.013)	–.278*** (.054)	.183*** (.039)	.300*** (.019)	–.058* (.032)
**Difference between CP Motive Effects**					
Firm-serving CP motives –					
Customer-serving CP motives ($\lvert\beta_{1j}\rvert - \lvert\beta_{2j}\rvert$)		–.260*** (.062)			–.039 (.036)

	DV = Firm-Serving CP Motives	DV = Customer-Serving CP Motives
	γ_{i1} (S.E.)	γ_{i2} (S.E.)
**Influence of CP Characteristics**		
CP design freedom (γ_{1j})	–.089*** (.032)	.309*** (.016)
CP intensity (γ_{2j})	.208*** (.014)	–.179*** (.012)

Notes: *p < .1; **p < .05; ***p < .01 (two-tailed tests).. Estimates show unstandardized coefficients; S.E. = Standard error; CP = Coproduction. Included control variables: Product category involvement; CP experience; CP situation; customer relationship length; DIY propensity; gender; age; income; reception of positive word of mouth and/or news coverage about focal company ($t_{0\text{-}5}$), reception of negative word of mouth and/or news coverage about focal company ($t_{0\text{-}5}$), positive experience with focal firm ($t_{0\text{-}5}$), negative experience with focal firm ($t_{0\text{-}5}$), indicator of whether a customer moved within the last 6 weeks ($t_{0\text{-}5}$). Standard errors of differences in effect sizes are based on multivariate delta method (e.g., Bishop, Fienberg, and Holland 1975).

Web Appendix F

Figure WF.1

Study 1 – Exemplary Within-Subject Means Based on Individual Growth Trajectories

A. Customers with Strong Customer-Serving and Weak Firm-Serving Coproduction Motive Attributions

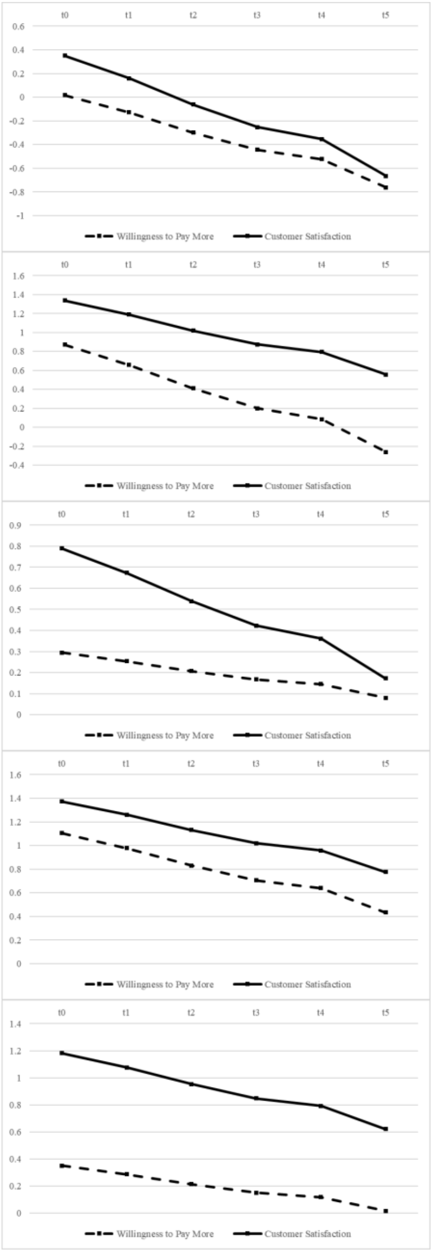

B. Customers with Strong Firm-Serving and Weak Customer-Serving Coproduction Motive Attributions

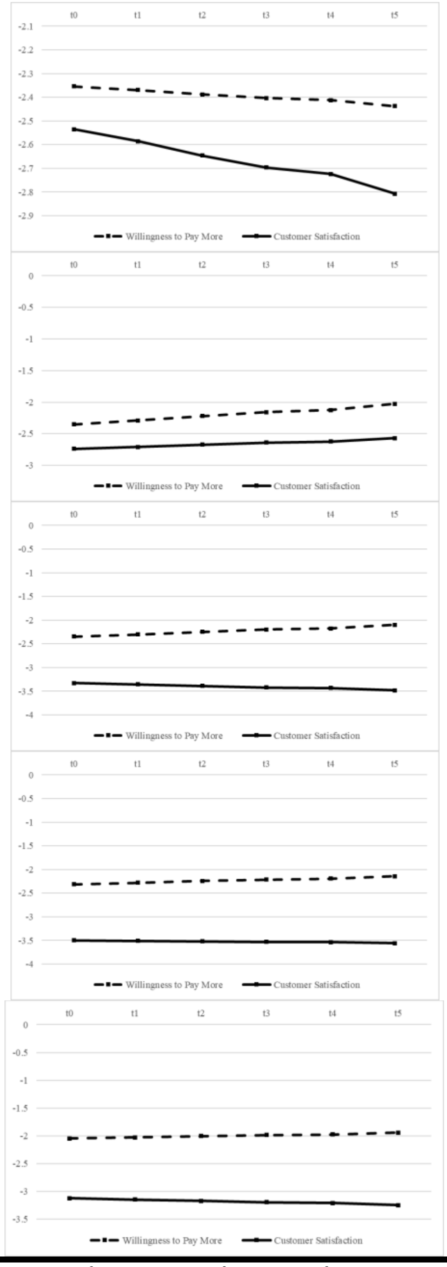

Notes: Strong attributions >= 6; weak attributions <= 2; latent means for customer satisfaction range from -3.8 to 1.7; latent means for customer satisfaction range from -2.5 to 3.7.

Figure WF.2

Study 1 – Development of the Effect of Firm- and Customer-Serving CP Motive Attributions on the Slope Parameters of the Customer Satisfaction and Willingness to Pay More Trajectories

A. Customer Satisfaction

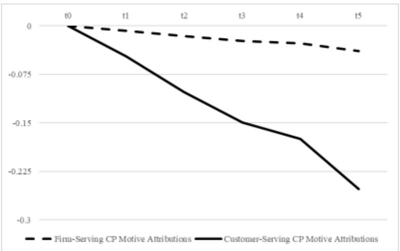

B. Willingness to Pay More

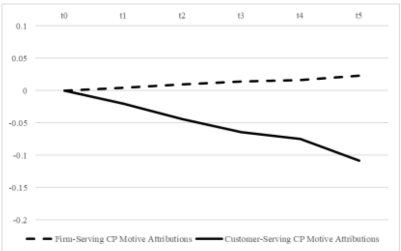

Notes: Plot based on model results in Table2; $((\beta_{i2} * \lambda_t)X_i)$, i = 1 for firm-serving and i = 2 for customer-serving CP motive attributions. Plotted for high values of both motive CP attributions.

Web Appendix G

Additional Moderation Analysis with Customer Category Involvement

Table WG.1

Core Results of the Interaction Effect between Customer-Serving Motive Attribution and Customer Category Involvement on the Slope Parameter of the Willingness to Pay Trajectory

	DV = Willingness to Pay More	
	Slope Parameter	
	**β**	**(S.E.)**
**Influence of CP Motives**		
Firm-serving CP motives (β_1)	.023	(.024)
Customer-serving CP motives (β_2)	–.044	(.032)
Product category involvement (β_3)	–.074*	(.042)
Customer-serving CP motives x Product category involvement (β_4)	.056**	(.026)
Firm-serving CP motives x Product category involvement (β_5)	.023	(.024)
**Simple Slope Analysis**		
**A) Low product category involvement**		
Firm-serving CP motives (ω_{L1})	–.017	(.031)
Customer-serving CP motives (ω_{L2})	–.100***	(.037)
Firm-serving CP motives – Customer-serving CP motives ($\|\omega_{L1}\| - \|\omega_{L2}\|$)	–.083*	(.049)
**B) High product category involvement**		
Firm-serving CP motives (ω_{H1})	.028	(.032)
Customer-serving CP motives (ω_{H2})	.012	(.045)
Firm-serving CP motives – Customer-serving CP motives ($\|\omega_{H1}\| - \|\omega_{H2}\|$)	.016	(.055)

Notes: n = 12,662; *p < .1; **p < .05; ***p < .01 (two-tailed tests). Estimates show unstandardized coefficients; S.E. = Standard error; CP = Coproduction. Influences of additional control variables not presented for ease of interpretation. Additionally included control variables: Product category involvement; CP experience; CP situation; customer relationship length; DIY propensity; gender; age; income; reception of positive word of mouth and/or news coverage about focal company (t_{0-5}), reception of negative word of mouth and/or news coverage about focal company (t_{0-5}), positive experience with focal firm (t_{0-5}), negative experience with focal firm (t_{0-5}), indicator of whether a customer moved within the last 6 weeks (t_{0-5}).

Web Appendix H

Ambivalence and Dominance of Coproduction Motive Attributions

Table WH.1

Study 1: Core Results of Dual-Process Latent Growth Analyses – Ambivalence and Dominance of Coproduction Motive Attributions

	Main Effects Model			
	DV = Customer Satisfaction		DV = Willingness to Pay More	
	Intercept β_{i1} (S.E.)	Slope β_{i2} (S.E.)	Intercept β_{i3} (S.E.)	Slope β_{i4} (S.E.)
**Dominance of CP Motives**				
CP motive dominance – extent (β_{1j})	–.075*** (.007)	.0001 (.011)	–.105*** (.011)	.017 (.019)
CP motive dominance – direction (β_{2j})	–.013 (.014)	.043* (.023)	–.074*** (.025)	.044 (.041)
CP motive dominance – direction x CP motive dominance – extent (β_{3j})	–.155*** (.008)	.009 (.013)	–.174*** (.013)	–.001 (.022)
**Simple Slopes**				
Firm-serving > Customer Serving CP motive dominance (ω_{1j})	–.230*** (.014)	-	–.279*** (.015)	-
Customer-serving > Customer Serving CP motive dominance (ω_{2j})	.080*** (.007)	-	.070*** (.018)	-

Notes: n = 12,662; *p < .1; **p < .05; ***p < .01 (two-tailed tests). Estimates show unstandardized coefficients; S.E. = Standard error; CP = Coproduction. Influences of additional control variables not presented for ease of interpretation. Additionally included control variables: Product category involvement; CP experience; CP situation; customer relationship length; DIY propensity; gender; age; income; reception of positive word of mouth and/or news coverage about focal company (t_{0-5}), reception of negative word of mouth and/or news coverage about focal company (t_{0-5}), positive experience with focal firm (t_{0-5}), negative experience with focal firm (t_{0-5}), indicator of whether a customer moved within the last 6 weeks (t_{0-5}).

Table WH.2

Study 2: Results of Experimental Study – Ambivalence and Dominance of Coproduction Motive Attributions

	DV = Customer Satisfaction		DV = Willingness to Pay More	
	β_{i1}	(S.E.)	β_{i2}	(S.E.)
**Dominance of CP Motives**				
CP motive dominance – extent (β_{1j})	–.062	(.058)	–.087	(.071)
CP motive dominance – direction (β_{2j})	.077	(.077)	–.088	(.084)
CP motive dominance – direction x CP motive dominance – extent (β_{3j})	–.231***	(.059)	–.092	(.072)
**Simple Slopes**				
Firm-serving > Customer Serving CP motive dominance (ω_{1j})	–.292***	(.033)	–.179***	(.020)
Customer-serving > Customer Serving CP motive dominance (ω_{2j})	.169	(.112)	.004	(.142)

Notes: n = 931; **p* < .1; ***p* < .05; ****p* < .01 (two-tailed tests). Estimates show unstandardized coefficients; S.E. = Standard error; CP = Coproduction.

Table WH.3

Study 3: Results of Multi-Firm Field Study – Ambivalence and Dominance of Coproduction Motive Attributions

	DV = Customer Satisfaction		DV = Willingness to Pay More	
	β_{i1}	(S.E.)	β_{i2}	(S.E.)
**Dominance of CP Motives**				
CP motive dominance – extent (β_{1j})	–.015	(.031)	–.077	(.054)
CP motive dominance – direction (β_{2j})	–.035	(.062)	–.119	(.104)
CP motive dominance – direction x CP motive dominance – extent (β_{3j})	–.099***	(.035)	–.138**	(.062)
**Simple Slopes**				
Firm-serving > Customer Serving CP motive dominance (ω_{1j})	–.114**	(.057)	–.215***	(.077)
Customer-serving > Customer Serving CP motive dominance (ω_{2j})	.084***	(.032)	.061	(.087)

Notes: n = 360; **p* < .1; ***p* < .05; ****p* < .01 (two-tailed tests). Estimates show unstandardized coefficients; S.E. = Standard error; CP = Coproduction. Included controls: CP price reduction, CP intensity, CP design freedom, product category involvement, CP experience, DIY propensity, customer relationship length, CP situation, gender, age, income.

Web Appendix I

Additional Mediation Analysis

Table WI1

Core Results of Mediation Analysis: Direct and Indirect Effects of Coproduction Design Freedom and Coproduction Intensity on the Growth Trajectories

Relationship	Estimate	(S.E.)
**Effects on the intercept parameter of the customer satisfaction growth trajectory**		
CP design freedom → Customer-serving CP motives → Intercept customer satisfaction	.120***	(.008)
CP design freedom → Firm-serving CP motives → Intercept customer satisfaction	.004***	(.001)
CP design freedom → Intercept customer satisfaction	.157***	(.013)
CP Intensity → Customer-Serving CP motives → Intercept customer satisfaction	–.069***	(.005)
CP Intensity → Firm-serving CP motives → Intercept customer satisfaction	–.009***	(.001)
CP Intensity → Intercept customer satisfaction	–.109***	(.009)
**Effects on the intercept parameter of the willingness to pay more growth trajectory**		
CP design freedom → Customer-serving CP motives → Intercept willingness to pay more	.093***	(.008)
CP design freedom → Firm-serving CP motives → Intercept willingness to pay more	.014***	(.003)
CP design freedom → Intercept willingness to pay more	.164***	(.022)
CP Intensity → Customer-serving CP motives → Intercept willingness to pay more	–.054***	(.005)
CP Intensity → Firm-serving CP motives → Intercept willingness to pay more	–.033***	(.004)
CP Intensity → Intercept willingness to pay more	–.073***	(.016)
**Effects on the slope parameter of the customer satisfaction growth trajectory**		
CP design freedom → Customer-serving CP motives → Slope customer satisfaction	–.039***	(.007)
CP design freedom → Firm-serving CP motives → Slope customer satisfaction	.001	(.001)
CP design freedom → Slope customer satisfaction	.018	(.020)
CP Intensity → Customer-serving CP motives → Slope customer satisfaction	.023***	(.004)
CP Intensity → Firm-serving CP motives → Slope customer satisfaction	–.003	(.002)
CP Intensity → Slope customer satisfaction	.011	(.015)
**Effects on the slope parameter of the willingness to pay more growth trajectory**		
CP design freedom → Customer-serving CP motives → Slope willingness to pay more	–.017*	(.010)
CP design freedom → Firm-serving CP motives → Slope willingness to pay more	–.001	(.002)
CP design freedom → Slope willingness to pay more	.039	(.034)
CP Intensity → Customer-serving CP motives → Slope willingness to pay more	.010*	(.006)
CP Intensity → Firm-serving CP motives → Slope willingness to pay more	.002	(.004)
CP Intensity → Slope willingness to pay more	.022	(.026)

Notes: n = 12,662; *p < .1; **p < .05; ***p < .01 (two-tailed tests). Estimates show unstandardized coefficients; S.E. = Standard error; CP = Coproduction. Influences of additional control variables not presented for ease of interpretation. Additionally included control variables: Product category involvement; CP experience; CP situation; customer relationship length; DIY propensity; gender; age; income; reception of positive word of mouth and/or news coverage about focal company ($t_{0\text{-}5}$), reception of negative word of mouth and/or news coverage about focal company ($t_{0\text{-}5}$), positive experience with focal firm ($t_{0\text{-}5}$), negative experience with focal firm ($t_{0\text{-}5}$), indicator of whether a customer moved within the last 6 weeks ($t_{0\text{-}5}$).

Web Appendix J

Additional Latent Growth Analyses Controlling for Respondent Attrition

Table WJ.1

Core Results of Dual-Process Latent Growth Analyses with Pattern Mixture-Dropout Modeling

	Main Effects Model			
	DV = Customer Satisfaction		DV = Willingness to Pay More	
	**Intercept β (S.E.)**	**Slope β (S.E.)**	**Intercept β (S.E.)**	**Slope β (S.E.)**
**Influence of CP Motives**				
Firm-serving CP motives (β_1)	–.041*** (.006)	–.018 (.012)	–.157*** (.013)	.008 (.021)
Customer-serving CP motives (β_2)	.387*** (.013)	–.129*** (.021)	.300*** (.019)	–.053* (.032)
**Difference between CP Motive Effects**				
Firm-serving CP motives – Customer-serving CP motives ($\lvert\beta_1\rvert - \lvert\beta_2\rvert$)		–.111*** (.023)		–.045 (.036)
**Effects of Pattern Mixture Dropout Indicators**				
Dropout after t = 0	.021 (.020)	–.016 (.012)	–.113*** (.037)	.146*** (.024)
Dropout after t = 1	–.010 (.034)	–.484*** (.149)	–.170*** (.059)	.449* (.264)
Dropout after t = 2	.059** (.028)	–.335*** (.062)	–.148** (.057)	–.145 (.121)
Dropout after t = 3	.062** (.028)	–.267*** (.047)	–.078 (.052)	.064 (.079)
Dropout after t = 4	.040* (.026)	–.173*** (.037)	–.101** (.050)	.308*** (.062)

	DV = Firm-Serving CP Motives	DV = Customer-Serving CP Motives
	**γ (S.E.)**	**γ (S.E.)**
**Influence of CP Strategies**		
CP design freedom (γ_1)	–.089*** (.021)	.310*** (.018)
CP intensity (γ_2)	.208*** (.014)	–.179*** (.012)

Notes: n = 12,662; **p* < .1; ***p* < .05; ****p* < .01 (two-tailed tests). Estimates show unstandardized coefficients; S.E. = Standard error; CP = Coproduction. Standard errors of differences in effect sizes are based on multivariate delta method (e.g., Bishop, Fienberg, and Holland 1975). Influences of additional control variables not included for reasons of clarity and ease of interpretation. Additional controls: Product category involvement; CP experience; CP situation; customer relationship length; DIY propensity; gender; age; income; reception of positive word of mouth and/or news coverage about focal company ($t_{0\text{-}5}$), reception of negative word of mouth and/or news coverage about focal company ($t_{0\text{-}5}$), positive experience with focal firm ($t_{0\text{-}5}$), negative experience with focal firm ($t_{0\text{-}5}$), indicator of whether a customer moved within the last 6 weeks ($t_{0\text{-}5}$).

Web Appendix K

Study 1 – Additional Robustness Checks

To further strengthen the robustness of our findings we conducted multiple additional robustness checks. To assess whether respondents' gender affects our findings, we conducted two additional analyses in which gender moderates a) the effects of coproduction characteristics on coproduction motive attributions and b) the effects of coproduction motive attributions on customer outcomes. Results remain stable irrespective of including these interaction effects.

Whereas we included a measure for general customer coproduction experience (at t = 0) in our model, this variable does not account for coproduction experiences across waves. We therefore included two time-varying covariates (t = 0 – 5) in the main model to account for positive and negative experiences across waves. Results remain stable irrespective of including these variables in the model. In addition, we conducted an additional analysis in which we included another dummy-coded time-varying covariate in the model (t = 0 – 5), which captures whether the customer has purchased something from the company in the respective wave. Results remain stable irrespective of including these additional control variables.

Although our reasoning in developing H_1-H_4 does not directly relate to a specific coproduction event, it is important to assess how such recent events may influence our findings. We therefore conducted two additional analyses. First, we estimated a model in which we included interaction effects between the time since the customers' last coproduction experience with the firm and both coproduction motive attributions on the growth trajectories of both customer outcomes. Results reveal only one significant interaction effect between time and firm-serving coproduction motive attribution on the intercept of the willingness to pay more trajectory ($\beta_{43} = –.001$, $p <.01$). Results of this model are in line with the results of the main model. Second, we estimated a model in which we included only customers who coproduced ready-to-assemble furniture from the company within the last 6 weeks. Results of this model are also in line with the results of the main model. Results of these additional analyses underline the robustness of our findings.

Web Appendix L

Additional Validation Analysis with Objective Data on Customer Spending

To validate our findings with objective data and to gain insights into the downstream consequences of inferred firm motives, we estimated an additional autoregressive model including firm data on customer spending[1]. This model includes the lagged effects of customer satisfaction and customer willingness to pay more on customer spending ($CS_t \rightarrow Spending_{t+1}$; $WTPM_t \rightarrow Spending_{t+1}$) as well as the autoregressive lagged effects of customer satisfaction, willingness to pay more, and customer spending ($CS_t \rightarrow CS_{t+1}$; $WTPM_t \rightarrow WTPM_{t+1;}$ $Spending_t \rightarrow Spending_{t+1}$). We also included the direct effects of firm- and customer-serving motive attributions on customer satisfaction, customer willingness to pay more, and customer spending in the model. Finally, we added the effects of coproduction design freedom and intensity on both motive attribution as well as all control variables from the main analyses in the model.

The results of this model show highly significant effects of customer satisfaction ($\beta_{2, CS} = 3.293$, $p < .05$) and willingness to pay more on customer spending ($\beta_{4, WTPM} = 2.138$, $p < .01$) (see Web Appendix M). Furthermore, we find significant total indirect effects of firm- and customer-serving motive attributions on customer spending via customer outcomes ($\beta_{IE1, Firm\text{-}serving} = –.731$, $p < .01$; $\beta_{IE2, Customer\text{-}serving} = 2.547$, $p < .01$). Finally, we also find significant total indirect effects of coproduction design freedom and intensity on customer spending via both motive attributions and customer outcomes ($\beta_{IE3, DesignFreedom} = 1.639$, $p < .01$; $\beta_{IE4, CoproductionIntensity} = –1.073$, $p < .01$).[2]

[1] The focal firm provided objective data on individual customer spending. Note that we employed an autoregressive model for this additional analysis, because a latent growth modeling approach does not allow the simultaneous estimation of a conditional latent growth model and the direct effects of the constituent of the growth trajectories (i.e., CS_t and $WTPM_t$) on another outcome variable (*here:* customer spending).

[2] In light of the direct effects (see Table WL.1), we thus find support for full mediation for the effects of firm-serving coproduction motive attribution, CP design freedom, and intensity on customer spending and partial mediation for customer-serving coproduction motive attribution.

Table WL.1

Core Results of Autoregressive Structural Equation Analysis with Objective Data on Customer Spending

Relationship		Estimate	(S.E.)
**Lagged and Cross-Lagged Effects – Autoregressive Model Part**			
Customer satisfaction$_{(t)}$ → Customer satisfaction$_{(t+1)}$	(β_1)	.809***	(.008)
Customer satisfaction$_{(t)}$ → Customer spending$_{(t+1)}$	(β_2)	3.293**	(1.297)
Willingness to pay more$_{(t)}$ → Willingness to pay more$_{(t+1)}$	(β_3)	.735***	(.008)
Willingness to pay more$_{(t)}$ → Customer spending$_{(t+1)}$	(β_4)	2.138***	(.733)
Customer spending$_{(t)}$ → Customer spending$_{(t+1)}$	(β_5)	.110***	(.015)
**Direct Effects of CP Motive Attributions**			
Firm-serving CP motives → Customer satisfaction	(β_6)	–.039***	(.006)
Firm-serving CP motives → Willingness to pay more	(β_7)	–.156***	(.013)
Firm-serving CP motives → Customer spending	(β_8)	–2.444	(1.930)
Customer-serving CP motives → Customer satisfaction	(β_9)	.393***	(.014)
Customer-serving CP motives → Willingness to pay more	(β_{10})	.297***	(.019)
Customer-serving CP motives → Customer spending	(β_{11})	5.616**	(2.165)
**Direct Effects of CP Strategies**			
CP design freedom → Firm-serving CP motives	(γ_1)	–.088***	(.021)
CP design freedom → Customer-serving CP motives	(γ_2)	.310***	(.018)
CP design freedom → Customer spending	(γ_3)	-.343	(2.963)
CP intensity → Firm-serving CP motives	(γ_4)	.209***	(.014)
CP intensity → Customer-serving CP motives	(γ_5)	–.179***	(.012)
CP intensity → Customer spending	(γ_6)	.372	(2.206)
**Total Indirect Effects of CP Motive Attributions on Customer Spending** (t_0 → t_1)			
Firm-serving CP motives → Customer spending	(β_{IE1})	–.731***	(.244)
Customer-serving CP motives → Customer spending	(β_{IE2})	2.547***	(.528)
**Total Indirect Effects of CP Characteristics on Customer Spending** (t_0 → t_1)			
CP design freedom → Customer spending	(β_{IE3})	1.639***	(.463)
CP intensity → Customer spending	(β_{IE4})	–1.073***	(.325)

Notes: n = 12,662; *p < .1; **p < .05; ***p < .01 (two-tailed tests). Estimates show unstandardized coefficients; S.E. = Standard error; CP = Coproduction. Influences of additional control variables not presented for ease of interpretation. Additionally included control variables: Product category involvement, CP experience, customer relationship length, DIY propensity, CP situation (coproduced for oneself or others), age, gender, income, reception of positive word of mouth and/or positive press release about focal company (t = 0 – 5), reception of negative word of mouth and/or negative press release about focal company (t = 0 – 5), positive experience with focal company (t = 0 – 5), negative experience with focal company (t = 0 – 5); moved within the last 6 weeks (t = 0 – 5). Note that we followed established procedures in autoregressive path modeling and constrained lagged and cross-lagged effects over time (e.g., Cole and Maxwell 2003; Little et al. 2007).

Additional References of the Web Appendix

Biesanz, Jeremy C., Natalia Deeb-Sossa, Alison A. Papadakis, Kenneth A. Bollen, and Patrick J. Curran (2004). The role of coding time in estimating and interpreting growth curve models. *Psychological Methods*, 9 (1), 30-52.

Bentein, Kathleen, Christian Vandenberghe, Robert Vandenberg, and Florence Stinglhamber, (2005). The role of change in the relationship between commitment and turnover: a latent growth modeling approach. *Journal of Applied Psychology*, 90(3), 468-482.

Bishop, Yvonne M. M., Stephen E. Fienberg, and Paul W. Holland (1975). *Discrete Multivariate Analysis: Theory and Practice*. Cambridge, MA: The MIT Press.

Bollen, Kenneth A. and P. J. Curran (2006). *Latent curve models: A structural equation approach*. Hoboken, NJ: Wiley.

Chan, David (1998). Functional Relations Among Constructs in the Same Content Domain at Different Levels of Analysis: A Typology of Composition Models. *Journal of Applied Psychology*, 83 (2), 234–246.

Cole, David A. and Scott E. Maxwell (2003). Testing mediational models with longitudinal data: questions and tips in the use of structural equation modeling. *Journal of Abnormal Psychology*, 112 (4), 558–577.

Grayson, Kent (2007). Friendship Versus Business in Marketing Relationships. *Journal of Marketing*, 71 (4), 121–139.

Griffith, David A. and Robert F. Lusch (2007). Getting Marketers to Invest in Firm-Specific Capital. *Journal of Marketing*, 71 (1), 129–145.

Kline, Rex B. (2015). *Principles and Practice of Structural Equation Modeling*. Fourth edition. New York: The Guilford Press.

Lindell, Michael K. and David J. Whitney (2001). Accounting for Common Method Variance in Cross-sectional Research Designs. *Journal of Applied Psychology*, 86 (1), 114–121.

Little, Todd D., Kristopher J. Preacher, James P. Selig, and Noel A. Card (2007) . New developments in latent variable panel analyses of longitudinal data. *International Journal of Behavioral Development*, 31 (4), 357–365.

Olson, Eric M., Stanley F. Slater, and G. Tomas M. Hult (2005).The performance implications of fit among business strategy, marketing organization structure, and strategic behavior. *Journal of Marketing*, 69 (3), 49–65.

Ployhart, Robert E. and Robert J. Vandenberg (2010). Longitudinal research: The theory, design, and analysis of change. *Journal of Management*, 36 (1), 94–120.

Podsakoff, Philip M., Scott B. MacKenzie, Jeong-Yeon Lee, and Nathan P. Podsakoff (2003). Common method biases in behavioral research: A critical review of the literature and recommended remedies. *Journal of Applied Psychology*, 88 (5), 879–903.

Preacher, Kristopher J., Aaron L. Wichman, Robert C. MacCallum, and Nancy E. Briggs (2008). *Latent growth curve modeling*. Sage: Thousand Oaks.

Sayer, Aline G., & Patricio E. Cumsille (2001). Second-order latent growth models. In Linda M. Collins & Aline G. Sayer (Eds.), *New methods for the analysis of change* (pp. 179–200). American Psychological Association: Washington D.C.

Sanchez, Juan I., William P. Korbin, and Diana M. Viscarra (1995). Corporate support in the aftermath of a natural disaster: effects on employee strains. *Academy of Management Journal*, 38 (2), 504–521.

Sheng, Shibin, Kevin Z. Zhou, and Julie J. Li (2011). The effects of business and political ties on firm performance: Evidence from China. *Journal of Marketing*, 75 (1), 1–15.

Siemsen, Enno, Aleda Roth, and Pedro Oliveira (2010). Common Method Bias in Regression Models with Linear, Quadratic and Interaction Effects. *Organizational Research Methods*, 13 (3), 456–476.

Williams, Larry J., Jeffrey R. Edwards, and Robert J. Vandenberg (2003). Recent advances in causal modeling methods for organizational and management research. *Journal of Management*, 29 (6), 903-936.
